# Human SERPINA3 induces neocortical folding and improves cognitive ability in mice

**DOI:** 10.1038/s41421-022-00469-0

**Published:** 2022-11-22

**Authors:** Jinyue Zhao, Chao Feng, Wenwen Wang, Libo Su, Jianwei Jiao

**Affiliations:** 1 State Key Laboratory of Stem Cell and Reproductive Biology, Institute of Zoology, Chinese Academy of Sciences, Beijing, China; 2 Beijing Institute for Stem Cell and Regenerative Medicine, Institute for Stem Cell and Regeneration, Chinese Academy of Sciences, Beijing, China; 3 University of Chinese Academy of Sciences, Beijing, China; 4 Max Planck Institute of Neurobiology, Martinsried, Germany; 5 School of Life Sciences, University of Science and Technology of China, Hefei, China

**Keywords:** Developmental biology, Neural stem cells

## Abstract

Neocortex expansion and folding are related to human intelligence and cognition, but the molecular and cellular mechanisms underlying cortical folding remain poorly understood. Here, we report that the human gene *SERPINA3* is linked to gyrification. Specifically, the overexpression of SERPINA3 induced neocortical folding, increased the abundance of neurons, and improved cognitive abilities. Further, SERPINA3 promoted proliferation of the outer radial glia (oRG, also referred to as the basal radial glia) and increased the number of upper-layer neurons. The downstream target *Glo1* was determined to be involved in SERPINA3-induced gyrification. Moreover, SERPINA3 increased the proliferation of oRG by binding to the *Glo1* promoter. Assessment of behavior performance showed enhanced cognitive abilities in *SERPINA3* knock-in mice. Our findings will enrich the understanding of neocortical expansion and gyrification and provide insights into possible treatments for intellectual disability and lissencephaly syndrome.

## Introduction

Neocortex expansion during human evolution enables higher intellectual capacity and improved cognitive functions^1–4^. A key aspect of this expansion is the formation of gyrification, which allows enlargement of the cortical surface area to fit into a limited cranium space^1,5–7^. These evolutionary features reflect the higher number of cortical neurons generated by various neural stem and progenitor cell subtypes and their neurogenic divisions^8,9^. Recent studies have shown the emergence of an outer subventricular zone (oSVZ) in the primate cortex, consisting of a massive pool of proliferating outer radial glia (oRG), intermediate progenitors (IPs), and postmitotic neurons^1,8,9^. oRG cells are thought to be the main cause of cortical folding in gyrencephalic species^8–10^. In support of this hypothesis, several studies have demonstrated that in various species, oRG are associated with cortical folding. The downregulation of Trnp1 expression leads to the increased production of oRG and subsequent gyrification of the cerebral cortex^11^. Further, the overexpression of ARHGAP11B, TBC1D3, and TMEM14B promote the expansion of basal progenitors (BPs), resulting in gyrification of the cortex in the naturally lissencephalic mouse or gyrencephalic ferret^12–14^. However, both the cellular and molecular mechanisms that govern the development of oRG cells and the oSVZ, as well as the potential for those mechanisms to drive neocortical folding, are not fully understood^12,15–18^. In recent studies detecting gene expression characteristics in the developing neocortex, many evolutionary differences in gene expression were discovered between the smooth mouse neocortex and human fetal neocortex during embryonic development^16,19^. Genes that are expressed in the ventricular RG (vRG, also referred to as apical radial glia) and oRG in humans but not or lowly expressed in mouse during neurogenesis are more likely to be associated with cortical expansion^13,16,19^. We previously searched for an existing detailed single-cell transcriptome dataset of vRG, oRG, IP cells, and neurons^13^ and found that the human gene *SERPINA3* is one such potential candidate gene. Human SERPINA3 shares 61% homology with murine Serpina3n^20^. Moreover, SERPINA3 is highly expressed in radial glial cells^13^, but Serpina3n is not expressed in the brain during mouse neurodevelopment^21^. Hence, this result implies that SERPINA3 might be related to neocortical development. Most studies on SERPINA3 have been limited to its inhibitory functions and mechanisms with respect to serine proteases^22^, but some studies have shown that it also acts as a transcriptional regulator^23^. Herein, we report a new role of SERPINA3 in the transcriptional regulation of genes related to neurogenesis.

In this study, the overexpression of SERPINA3 in the embryonic mouse neocortex promoted the generation and self-renewal of BPs and further increased cortical thickness and induced gyrification. The increase in upper-layer neurons in the *SERPINA3*-conditional knock-in mouse cortex was confirmed by single-cell RNA sequencing (sc-RNA Seq). Furthermore, *SERPINA3*-conditional knock-in mice exhibited enhanced learning and memory abilities in behavior tests. These results indicated that the expression of human SERPINA3 in mice is involved in cortical expansion and cognitive enhancement. Intriguingly, we found that SERPINA3 acts as a transcription factor or cofactor to promote the expression of *Glo1*, which was previously found to be involved in the proliferation of BPs^24^.

## Results

### SERPINA3 is expressed in human radial glia cells

**Fig. 1:**
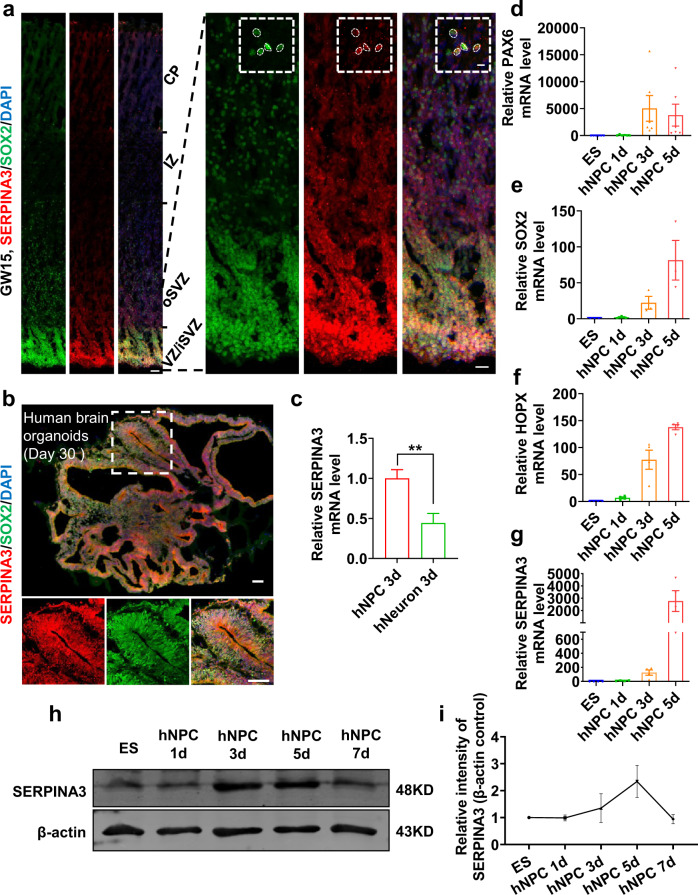
SERPINA3 is expressed in VZ/SVZ of human brain. **a** IF for SERPINA3 and SOX2 in GW15 human brain. Dashed square showed the enlarged part. Scale bars, 50 μm (left), 20 μm (bottom right) and 5 μm (top right). **b** IF for SERPINA3 and SOX2 in 30 days of brain organoids. Dashed square showed the enlarged part. Scale bars, 100 μm. **c** RT-qPCR was performed to detect the mRNA level of SERPINA3 in hNPCs and neurons cultured in vitro (*n* = 6). **d**–**g** RT-qPCR was performed to detect the relative mRNA level of PAX6 (*n* = 6), SOX2 (*n* = 3), HOPX (*n* = 4) and SERPINA3 (*n* = 4) in human ES cells and hNPCs cultured in vitro. **h** Western-blot analysis for the expression of SERPINA3 in human ES cells and cultured hNPCs in different time from day 1 to day 7. β-actin was detected as loading control. **i** Statistics of relative intensity of SERPINA3 (*n* = 3). Two-tail unpaired *t*-test is used to analyze the data. n.s. no significant difference; *P* < 0.05 (*), *P* < 0.01 (**), *P* < 0.001 (***).

To confirm the expression pattern of SERPINA3 in the human brain, we performed immunofluorescence staining (IF) for SERPINA3 and SOX2 at gestational week (GW) 15 in human brain sections (Fig. 1a). The result showed that SERPINA3 was expressed in the ventricular zone (VZ) and subventricular zone (SVZ) and that it colocalized with SOX2. Moreover, the subcellular localization of SERPINA3 in human neural progenitor cells (hNPCs) was mainly in the nucleus, which was validated by high resolution IF staining (Fig. 1a), consistent with that in a previous study^23^. This expression was also checked by IF for SOX2 and SERPINA3 in human brain organoids (day 30; Fig. 1b). We also performed RT-qPCR to check the expression of SERPINA3 in cultured hNPCs and neurons, which showed that NPCs had a higher SERPINA3 expression level than neurons (Fig. 1c). Further, we used RT-qPCR and western blot analysis to examine the expression level of SERPINA3 during the differentiation of cultured embryonic stem (ES) cells to hNPCs to mimic SERPINA3 expression changes during human brain development. The expression of SERPINA3 was increased with the development of ES cells to hNPCs and decreased at day 7, at which stage some NPCs started to differentiate in our system. The expression of PAX6 decreased and HOPX increased at day 5, which implied that most of the cultured cells might be converted to oRG at this time point (Fig. 1d–i).

### Ectopic expression of SERPINA3 expands the SVZ in mice

To explore the role of SERPINA3 during cortical development, we constructed an overexpression vector comprising *SERPINA3* fused with a 3× Flag tag. The SERPINA3 overexpression vector was transferred into NPCs located in the ventricle zone of the mouse neocortex through in utero electroporation (IUE) at embryonic day 13.5 (E13.5). SERPINA3 overexpression in the lissencephalic mouse brain increased the number of PAX6^+^GFP^+^ NPCs at E15.5 (Supplementary Fig. S1a, b). Moreover, we found that the overexpression of SERPINA3 increased the percentage of HOPX^+^GFP^+^ cells, which implied the generation of oRG (Supplementary Fig. S1c, d). The effect of SERPINA3 on proliferation was confirmed by the increase in the proportion of cells stained with the mitotic marker phosphorylated histone H3 (PH3) (Supplementary Fig. S1e, f). Following a 2 h bromodeoxyuridine (BrdU) pulse, we also found that the overexpression of SERPINA3 increased cellular proliferation, with more cells entering the S-phase (Supplementary Fig. S1g, h). Together, these data reveal that SERPINA3 might promote the proliferation of NPCs and the generation of oRG.

### *SERPINA3*-knock-in mice show enhanced neurogenesis and cortical folding

To further determine the effect of SERPINA3 expression on cerebral cortex development, we generated *SERPINA3*-floxed knock-in mice (Rosa26^+/fl-STOP-fl-SERPINA3^), which was driven by the CAG promoter. Floxed mice were then bred with Nestin-Cre mice to induce SERPINA3 expression in Nestin-positive cells and their progeny (Supplementary Fig. S2a; hereafter referred to as cKI^f/+^ mouse). Western blotting showed that human SERPINA3 was expressed in the cKI^f/+^ mouse brain (Supplementary Fig. S2b). Therefore, we successfully generated a conditional knock-in mouse line that overexpressed SERPINA3 protein in the neocortical NPCs.

**Fig. 2:**
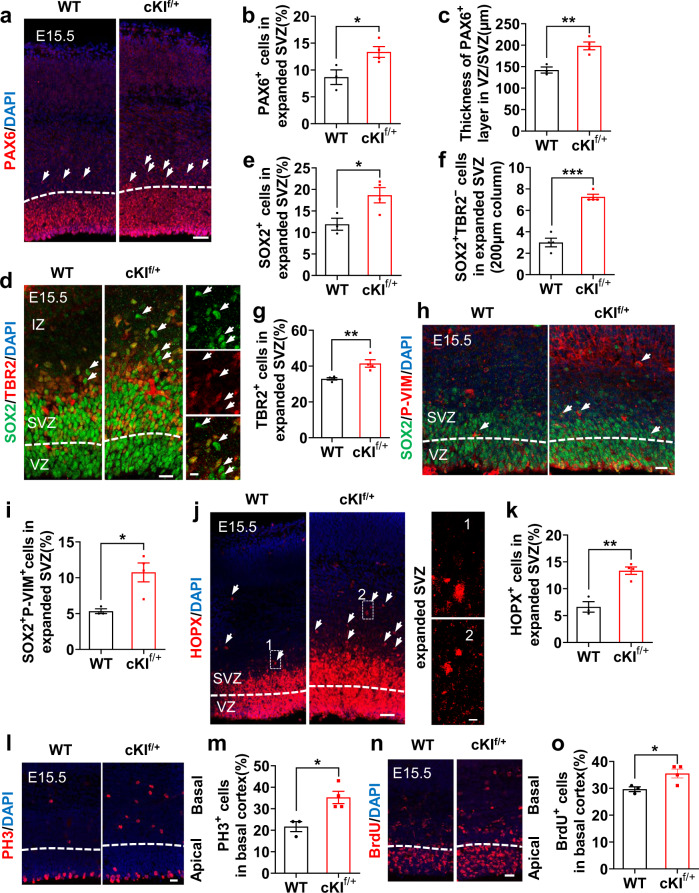
Conditional knock-in of human SERPINA3 leads to enhanced NPC proliferation and oRG generation in mice cortex. **a** IF for PAX6 in E15.5 WT and cKI^f/+^ mice. The dashed white lines represent the border of PAX6 layer in VZ/SVZ. Arrow, PAX6^+^ cells in expanded SVZ. Scale bar, 50 μm. **b** Quantification for the percentage of PAX6^+^ cells in the expanded SVZ (WT *n* = 3; cKI^f/+^ *n* = 4). **c** Quantification for the thickness of PAX6^+^ cells in VZ/SVZ (WT *n* = 3; cKI^f/+^ *n* = 4). **d** IF for SOX2 and TBR2 in E15.5 WT and cKI^f/+^ mice. The dashed white lines represent the border between VZ and SVZ. Arrow, SOX2^+^TBR2^−^ cells in expanded SVZ. Scale bars, 20 μm (left) and 10 μm (right). **e** Quantification for the percentage of SOX2^+^ cells in the expanded SVZ (WT *n* = 3; cKI^f/+^ *n* = 4). **f** Quantification for the number of SOX2^+^TBR2^−^ cells in the expanded SVZ (*n* = 4). **g** Quantification for the percentage of TBR2^+^ cells in the expanded SVZ (*n* = 4). **h** IF for SOX2 and P-VIM in E15.5 WT and cKI^f/+^ mice. Arrow, SOX2^+^P-VIM^+^ cells. Scale bar, 20 μm. **i** Quantification for the percentage of SOX2^+^ P-VIM^+^ cells in the expanded SVZ (WT *n* = 3; cKI^f/+^ *n* = 4). **j** IF for HOPX in the expanded SVZ of E15.5 WT and cKI^f/+^ mice. Images 1 and 2 show the expanded SVZ region. Arrow, HOPX^+^ cells. Scale bars, 50 μm (left) and 10 μm (right). **k** Quantification for the percentage of HOPX^+^ cells in the expanded SVZ (WT *n* = 3; cKI^f/+^ *n* = 4). **l** IF for PH3 in E15.5 WT and cKI^f/+^ mice. The dashed white lines represent the border between Apical and Basal. Scale bar, 20 μm. **m** Quantification for the percentage of PH3^+^ cells in basal cortex (WT *n* = 3; cKI^f/+^ *n* = 4). **n** IF for BrdU in E15.5 WT and cKI^f/+^ mice. The dashed white lines represent the border between Apical and Basal. Scale bar, 20 μm. **o** Quantification for the percentage of BrdU^+^ cells in basal cortex (WT *n* = 3; cKI^f/+^ *n* = 4). Two-tail unpaired *t*-test is used to analyze the data. n.s. no significant difference; *P* < 0.05 (*), *P* < 0.01 (**), *P* < 0.001 (***).

During wild-type (WT) mouse brain development, oRG are rare, and there is no distinct oSVZ^25^. However, the conditional expression of SERPINA3 in mouse NPCs resulted in cortical thickening at E15.5 (Supplementary Fig. S2c, d). We also found increased thickness of the VZ/SVZ with an increase in PAX6^+^ progenitors (Fig. 2a–c). In addition, we observed that the SOX2^+^ area expanded radially toward the intermediate zone (IZ) in the developing cKI^f/+^ mouse neocortex (Fig. 2d, e). The number of SOX2^+^TBR2^−^ and TBR2^+^ cells in the expanded SVZ also increased (Fig. 2f, g). In addition, SOX2^+^P-VIM^+^ NPCs in the expanded SVZ were increased in cKI^f/+^ mice (Fig. 2h, i). Moreover, we observed an increase in the percentage of HOPX^+^ cells in the expanded SVZ (Fig. 2j, k). PH3 staining and BrdU-pulsing analysis showed more actively mitotic cells in the basal cortex of cKI^f/+^ mice (Fig. 2l–o). It has been reported that a nonvertical (oblique or horizontal) cleavage-plane orientation, in relation to the ventricular surface of dividing vRG, increases the probability that daughter cells become oRG^26,27^. To explore whether SERPINA3 induces oRG generation, we investigated the cleavage plane angles of vRG. We observed that oblique divisions were increased in cKI^f/+^ vRG compared with those in WT cells, suggesting that SERPINA3 might promote oRG generation (Supplementary Fig. S2e, f). TUNEL staining of E15.5 embryo cerebral cortex slices showed no significant difference between WT and cKI^f/+^ mice. This result implied that SERPINA3 overexpression did not cause apoptosis (Supplementary Fig. S2g, h). These results suggest that SERPINA3 overexpression in mice results in an increase in BPs and an expansion of the SVZ.

**Fig. 3:**
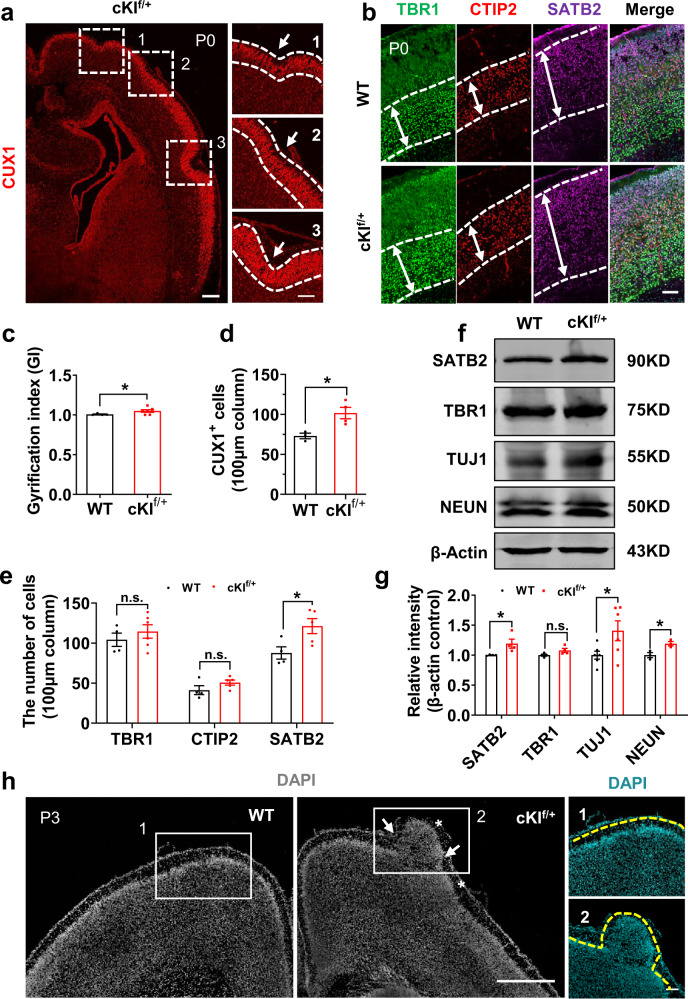
Human SERPINA3 overexpression in mouse NPCs induces cortical folding with expanded cortical lamination. **a** IF for CUX1 in P0 cKI^f/+^ brain section. Dashed square shows the curved cortex regions which were enlarged in the right side. Images 1, 2 and 3 are high-magnification images. Dashed white lines show the boundary of CUX1^+^ layer. Arrows indicate gyrification structures. Scale bars, 200 μm (left), 100 μm (right). **b** IF for TBR1, CTIP2 and SATB2 in P0 WT and cKI^f/+^ neocortex. The dashed white line and arrow show the thickness of positive layers. Scale bar, 50 μm. **c** Quantification for the GI of the cortex at P0 (WT *n* = 4; cKI^f/+^ *n* = 7). **d** Quantification for the CUX1^+^ cell number in 100 µm-wide field of cortex at P0 (WT *n* = 3; cKI^f/+^ *n* = 4). **e** Quantification for the TBR1^+^ cell number in 100 µm-wide field of cortex at P0 (WT *n* = 4; cKI^f/+^ *n* = 6). Quantification for the CTIP2^+^ cell number in 100µm-wide field of cortex at P0 (WT *n* = 4; cKI^f/+^ *n* = 5). Quantification for the SATB2^+^ cell number in 100 µm-wide field of cortex at P0 (WT *n* = 4; cKI^f/+^ *n* = 5). **f** Protein levels of neuron markers (SATB2, TBR1, TUJ1, and NEUN) in cortex of P0 WT and cKI^f/+^ were measured by western blotting. β-actin was detected as a loading control. **g** Quantification for the protein level of SATB2, TBR1, TUJ1, and NEUN in WT and cKI^f/+^ cortex (*n* = 4, 4, 6, and 3). **h** Staining for DAPI in P3 WT and cKI^f/+^ mice. Asterisks indicate gyrus and arrows indicate sulcus structures. White squares show the curved cortex region which were enlarged. Images 1 and 2 (DAPI) are high-magnification images. Scale bars, 500 μm (left) and 100 μm (right). Two-tail unpaired *t*-test is used to analyze the data. n.s. no significant difference; *P* < 0.05 (*), *P* < 0.01 (**), *P* < 0.001 (***).

To further investigate whether SERPINA3 expression can induce cortical expansion and gyrification, we examined the brains of postnatal day (P) 0 pups and found that the conditional expression of SERPINA3 led to a cerebral cortex with featured sulcus and gyrus (Fig. 3a and Supplementary Fig. S3a). We also found that approximately half of cKI^f/+^ mice showed apparent folding of the cortex at P0. The folding followed a random distribution and was mainly distributed in the frontal cortex. Next, we examined the structure of sulcus and gyrus by immunostaining for TUJ1, MAP2, and CTIP2 (Supplementary Fig. S3b, c). By comparing the WT and cKI^f/+^ cortexes, we observed that they both exhibited proper cortical lamination (Fig. 3b), but the cerebral cortex of cKI^f/+^ mice was enlarged and folded significantly, increasing the gyrification index (GI)^28–30^ from 1.0 (WT) to 1.05 (Fig. 3c). Then, to explore the effects of SERPINA3 on the formation of upper- and deep-layer neurons, we performed staining for SATB2, CTIP2, and TBR1 (Fig. 3b). We observed an increase in CUX1^+^ and SATB2^+^ upper-layer neurons in cKI^f/+^ mice, but not deep-layer CTIP2^+^ and TBR1^+^ neurons (Fig. 3d, e). Western blotting showed that the expression of SATB2, TUJ1, and NEUN was increased, whereas the expression of TBR1 did not change in the cortex (Fig. 3f, g). Considering all of these findings together, the number of upper-layer neurons is increased, but the number of deep-layer neurons is not affected by the overexpression of SERPINA3.

During the formation of these gyrifications and the rearrangement of neuronal layers, the cortical plate (CP) undergoes large morphological changes that could lead to the differential spacing of cells within the developing gyrus vs those in the sulcus^31^. Accordingly, we examined the nuclear distance within the CP neurons of WT and cKI^f/+^ mice. We observed a greater distance between neuronal nuclei in the gyrus than in the sulcus in cKI^f/+^ mice (Supplementary Fig. S3e, f). Cortical folding was also observed in P3 mice (Fig. 3h and Supplementary Fig. S3d), indicating that cortical folding was not temporary. Together, SERPINA3 expression in mouse NPCs leads to an increase in neurons, with proper lamination and cortical expansion and folding.

To further confirm the functions of SERPINA3 in neurogenesis, short hairpin RNA (shRNA) was used to knock down its expression in hNPCs. Immunostaining for SATB2 in differentiated human neurons revealed that the knockdown of *SERPINA3* led to a decrease in the percentage of SATB2^+^GFP^+^ cells (Supplementary Fig. S3g, h). Furthermore, western blotting showed that endogenous SERPINA3 was obviously reduced in *SERPINA3*-shRNA lentivirus-infected hNPCs and that the expression of CUX1 was decreased in differentiated neurons induced from *SERPINA3*-shRNA lentivirus-infected hNPCs (Supplementary Fig. S3i, j). These results suggest that a low level of SERPINA3 inhibits the generation of upper-layer neurons.

### Human SERPINA3 expression leads to an increase in layer II/III neurons

**Fig. 4:**
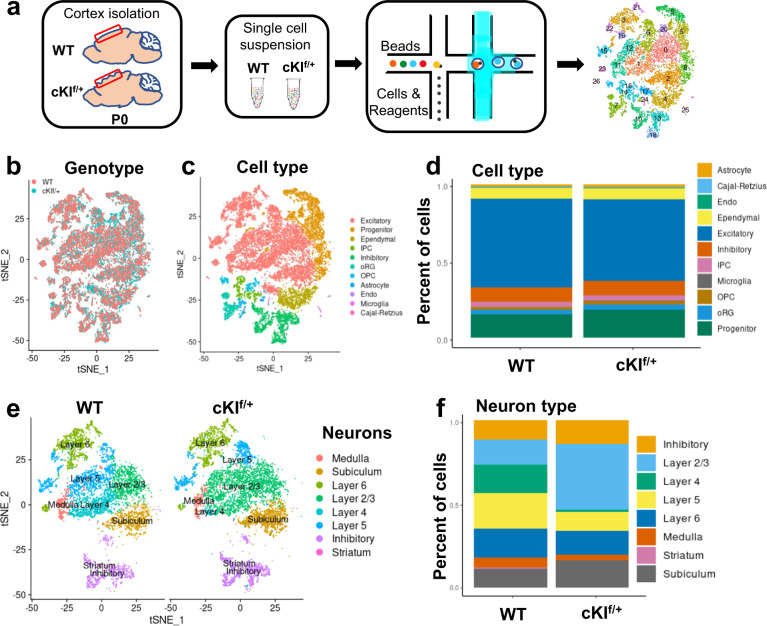
Sc-RNA Seq reveals that the overexpression of human SERPINA3 increases upper-layer neurons. **a** Pipeline of sc-RNA Seq processes with P0 WT and cKI^f/+^ cortex. **b** t-SNE plot for integrated WT and cKI^f/+^ transcriptome, red represents WT cells and blue represents cKI^f/+^ cells. **c** t-SNE plot for integrated WT and cKI^f/+^ transcriptome, each color shows a cell type cluster. Detail information of 11 cell types is on the right side. **d** Bar plot shows the ratio of each cell types. **e** t-SNE plot for different neuronal cell types. **f** Bar plot shows the ratio of each neuronal cell types.

To better understand the changes in neuronal diversity and cell type differences, we performed sc-RNA Seq of the cortex of P0 WT and cKI^f/+^ mice. The cortex was digested into single-cell suspensions and 10× single-cell gene expression analysis was performed as described in Fig. 4a. After pre-processing and removing mitochondrial genes, these two samples were integrated based on the Harmony algorithm, and 26 clusters were identified using the SEURAT package in the R platform (Supplementary Fig. S4a–c). The clusters were identified as 11 main cell types and distinguished by the top-ranked markers of each cell type (Supplementary Fig. S4d, e and Table S1). All information is shown in t-SNE plots (Fig. 4b, c). The ratio of different cell types was also calculated (Fig. 4d). Then, we analyzed the proportions of different neuronal cell types. Different clusters of neuronal cells were calculated through the unsupervised clustering method (Supplementary Fig. S4f). The supervised cell-type identification was based on the different cell-type marker genes published on the Allen Brain Atlas database. Here, the ratio of layer II/III neurons was increased in cKI^f/+^ mice (Fig. 4e, f). The increase in layer II/III neurons, from sc-RNA Seq analysis, was consistent with the results of P0 cortex immunostaining for markers of different layers. Moreover, we checked different glial cell markers, including Olig2 for oligodendrocytes and S100β, GFAP, and ALDH1L1 for astrocytes. No significant difference was observed, which implied that the overexpression of SERPINA3 did not affect gliogenesis (Supplementary Fig. S5a–f).

### Cortical expansion and increased upper-layer neuron numbers in the cKI^f/+^ mouse neocortex persist into adulthood

**Fig. 5:**
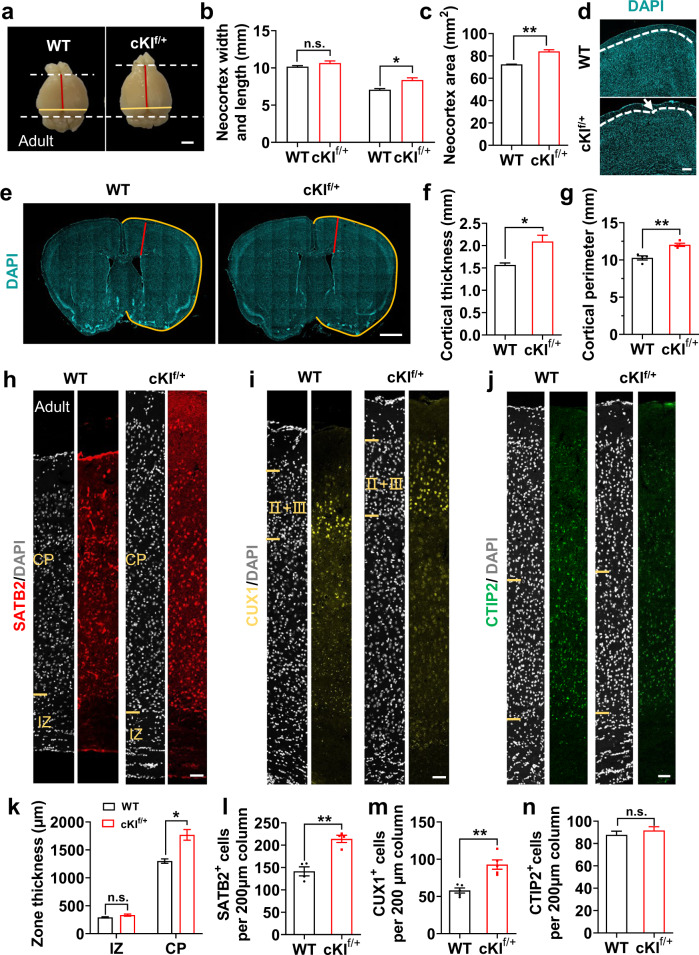
Persistence of neocortex expansion and increased cortical neuron numbers in adult cKI^f/+^ mice. **a** Dorsal view of adult WT and cKI^f/+^ mouse brain. Orange and red lines illustrate the measurements of neocortex width and length. Scale bar, 2 mm. **b**, **c** Quantification for neocortex width, length and area (*n* = 3). **d** DAPI staining of adult WT and cKI^f/+^ mouse neocortex. Arrows indicate gyrification structures. The dashed white lines show the boundary of cortex. **e** Representative DAPI staining of adult WT and cKI^f/+^ mouse neocortex illustrating the measurements of cortical thickness (red) and perimeter (orange). Scale bar, 1 mm. **f**, **g** Quantification for cortical thickness (*n* = 3) and perimeter (*n* = 4) in the rostral part of adult WT and cKI^f/+^ mouse brain. **h–j** IF for SATB2, CUX1 and CTIP2, combined with DAPI staining of adult WT and cKI^f/+^ mouse neocortex. Scale bars, 50 μm. **k** Quantification for zone thickness of adult WT and cKI^f/+^ mouse neocortex (*n* = 3). **l**–**n** Quantification for SATB2^+^, CUX1^+^ and CTIP2^+^ neurons in a 200 µm-wide field of adult WT and cKI^f/+^ mouse neocortex (*n* = 4, 5, and 3). Two-tail unpaired *t*-test is used to analyze the data. n.s. no significant difference; *P* < 0.05 (*), *P* < 0.01 (**), *P* < 0.001 (***).

It has recently been reported that the cortical expansion and increase in upper-layer neurons observed in transgenic mice expressing the human-specific *ARHGAP11B* gene persists into adulthood^14^, which can facilitate an investigation of cognitive abilities in these mice. The conditional *SERPINA3*-knock-in mouse line expressing SERPINA3 in the neocortex allowed us to extend our studies to the adult stage. Key aspects of neocortex expansion and cortical neuron increases observed in P0 cKI^f/+^ mice were found to persist into adulthood. We observed an increase in the neocortex length and area in adult cKI^f/+^ mice (Fig. 5a–c). Cortical folding was also observed in the adult cKI^f/+^ mice, although the curved region in the adult mouse brain was not obvious as pups (Fig. 5d and Supplementary Fig. S5g). Moreover, the increase in the cortical thickness and perimeter was observed in adult cKI^f/+^ mice (Fig. 5e–g), especially the increase in the CP thickness (Fig. 5h, k). The adult cKI^f/+^ mouse neocortex showed increased numbers of SATB2^+^ and CUX1^+^ upper-layer neurons (Fig. 5h, i, l, and m), but not CTIP2^+^ deep-layer neurons (Fig. 5j, n). We also found that there was no significant difference in the MBP level in oligodendrocytes in the adult brain, indicating that the overexpression of SERPINA3 might have no effect on the white matter of adult brains (Supplementary Fig. S5h, i).

### Human SERPINA3 improves learning and memory ability in adult mice

**Fig. 6:**
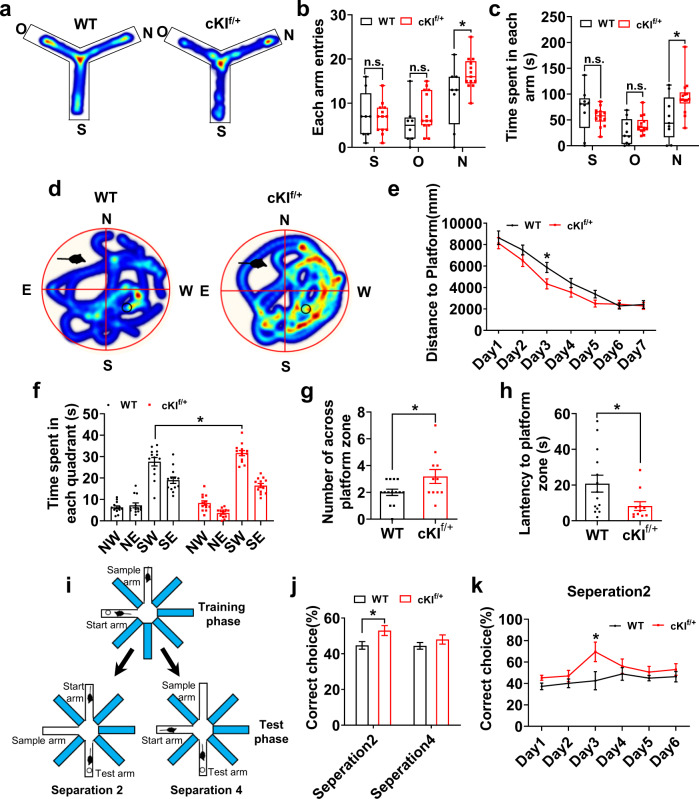
Human SERPINA3 overexpression may improve learning and memory in mice. **a** Representative trace track of WT and cKI^f/+^ mice in Y-maze test. Start arm(S), Old arm(O), New arm(N). **b** Quantification for the entries of each arm between WT and cKI^f/+^ mice in the Y-maze test (WT *n* = 8; cKI^f/+^ *n* = 13). **c** Quantification for the time spent in each arm between WT and cKI^f/+^ mice in the Y-maze test (WT *n* = 9; cKI^f/+^ *n* = 14). **d** Representative tracing path of WT and cKI^f/+^ mice in Morris water maze test. **e** Quantification for the distance to the platform during the training process (*n* = 13). **f** Quantification for time spent in each of the four quadrants of the water maze after the platform was removed following the initial 7 days of training (*n* = 13). **g** Quantification for number of across platform zone (WT *n* = 14; cKI^f/+^ *n* = 11). **h** Quantification for latency to platform zone (WT *n* = 14; cKI^f/+^ *n* = 11). **i** Schematic drawing of the DNMP-RAM test. **j** Quantification for the percentage of correct choice in separation 2 test and separation 4 test (WT *n* = 16; cKI^f/+^ *n* = 12). **k** Quantification for the percentage of correct choice between WT and cKI^f/+^ mice in separation 2 test (WT *n* = 15; cKI^f/+^ *n* = 11). Two-tail unpaired *t*-test is used to analyze the data. n.s. no significant difference. *P* < 0.05 (*), *P* < 0.01 (**), *P* < 0.001 (***).

Cortical expansion and folding usually lead to the evolution of higher intelligence and learning ability^12,31,32^. Because SERPINA3 expression resulted in cortical expansion and folding, we then tested whether SERPINA3 expression would affect neurobehavior, such as learning and memory abilities. The survival rate of adult WT and cKI^f/+^ mice was 100%, which showed that SERPINA3 did not affect mouse overall health (Supplementary Fig. S6a). We then tested the locomotor ability of mice using the open field test. We found that there was no apparent difference in time spent in the center area, total distance traveled or velocity between cKI^f/+^ and WT mice (Supplementary Fig. S6b–e). These results suggested no apparent difference in motility. Next, we performed the elevated-plus maze test to determine whether cKI^f/+^ mice show anxiety-like behavior. There was no significant difference in the time spent in the open arms and closed arms (Supplementary Fig. S6f–h). These results suggested that cKI^f/+^ mice had no anxiety-like behaviors. To evaluate the learning and memory ability of cKI^f/+^ mice, Y-maze tests and novel object recognition (NOR) tests were performed (Fig. 6a and Supplementary Fig. S6i). The results showed that cKI^f/+^ mice were more prone to enter new arms and spent more time in the new arms, indicating that working memory was improved (Fig. 6b, c). The NOR test also showed that cKI^f/+^ mice had a greater preference for novel objects (Supplementary Fig. S6j), suggesting that the memory of cKI^f/+^ mice was improved.

To further test whether the expression of SERPINA3 affects spatial learning and memory, cKI^f/+^ and WT mice were examined by performing the Morris water maze experiment. During the training phase, cKI^f/+^ mice spent less time reaching the platform than WT mice, starting from day 2 and becoming steady at approximately day 5 (Fig. 6d, e). The probe test was performed 24 h after the last training. The cKI^f/+^ mice spent more time in the target quadrant than the WT mice (Fig. 6f). Moreover, both the number of platform crossings and the time spent in the target platform zone were increased in cKI^f/+^ group compared with those in WT group when the hidden platform was removed (Fig. 6g, h). Together, these results suggest that cKI^f/+^ mice have increased ability for spatial learning and memory.

Pattern separation is a difficult form of spatial learning and memory. To determine whether SERPINA3 expression improves pattern separation, cKI^f/+^ mice and their WT littermates were subjected to a radial eight-arm maze pattern separation experiment (Fig. 6i). In this test, cKI^f/+^ mice made more correct choices in the one arm (separation 2, easy task) task compared with WT mice, and in the three arm (separation 4, hard task) task, cKI^f/+^ mice had a greater tendency to make the correct choice than WT mice, but without a significant difference (Fig. 6j, k). These results demonstrated that cKI^f/+^ mice show an improvement in pattern separation. To further confirm that the improvement in cKI^f/+^ mouse learning and memory was not caused by the effects of the hippocampus, we performed BrdU labeling in adult mice. The results showed that there was no significant difference in the proliferation of hippocampus cells between adult WT and cKI^f/+^ mice (Supplementary Fig. S6k, l).

### SERPINA3 regulates the proliferation of NPCs through Glo1 to induce cortical expansion and folding

**Fig. 7:**
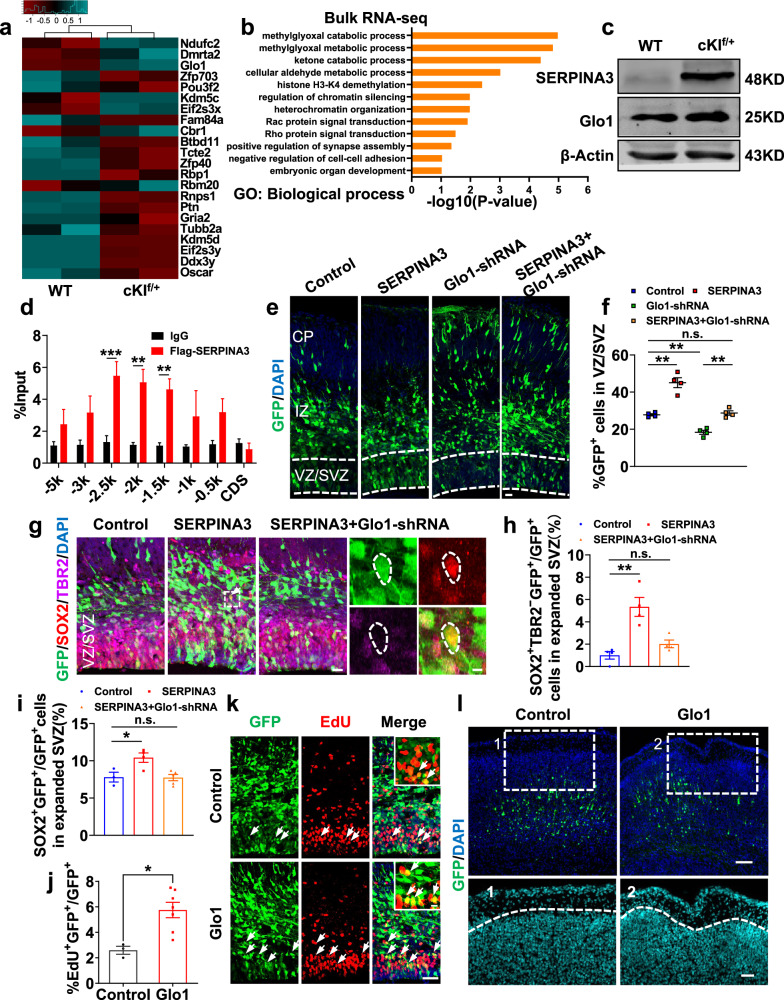
SERPINA3 regulates cortical expansion through Glo1. **a** Differential representation heatmap of the different expression genes. **b** Gene ontology analysis for regulated genes based on RNA-seq data in E15 cKI^f/+^ mice. **c** Western blot analysis for Glo1 in cortex of WT and cKI^f/+^ mice. β-actin was detected as loading control. **d** ChIP-qPCR analysis for SERPINA3 binding region in the *Glo1* promoter (*n* = 3). **e** GFP^+^ cell distribution in E15.5 mice after IUE at E13.5. Scale bar, 20 μm. **f** Quantification for the percentage of GFP^+^ cells in VZ/SVZ (*n* = 4). **g** IF for SOX2 and TBR2 in E15.5 mice after IUE at E13.5. Outlined by dashed white lines, SOX2^+^TBR2^−^ cells in expanded SVZ. Scale bars, 20 μm (left), 5 μm (right). **h** Quantification for the percentage of SOX2^+^TBR2^−^ cells among GFP^+^ cells in expanded SVZ (*n* = 4). **i** Quantification for the percentage of SOX2^+^ cells among GFP^+^ cells in expanded SVZ (control *n* = 3; SERPINA3 *n* = 4; SERPINA3 + Glo1-shRNA *n* = 5). **j** Quantification for the percentage of EdU^+^ cells among GFP^+^ cells (control *n* = 3; Glo1 *n* = 7). **k** IF for EdU in E15.5 mice after IUE at E13.5. Higher magnification images show the EdU^+^GFP^+^ cell (arrow, top right). Scale bars, 50 μm, 10 μm (top right). **l** Staining for DAPI in P0 mice after IUE at E13.5. Images 1 and 2 (DAPI) are high-magnification images. Dashed white lines show the boundary of cortex. Scale bars, 100 μm (top), 50 μm (bottom). Two-tail unpaired *t*-test is used to analyze the data. n.s. no significant difference; *P* < 0.05 (*), *P* < 0.01 (**), *P* < 0.001 (***).

To gain deeper insight into how SERPINA3 regulates embryonic neurogenesis, bulk RNA sequencing (bulk-RNA-seq) was performed to analyze the genome-wide changes in the cortices of E15 cKI^f/+^ mice and WT littermate controls. The differentially expressed genes are shown in a heatmap (Fig. 7a). Gene ontology analysis showed that the upregulated genes were related to biological processes such as the methylglyoxal (MG) catabolic process and metabolic process (Fig. 7b). It has been reported that increased MG expression suppresses notch signaling and leads to the depletion of NPCs in the developing mouse cortex^33,34^. Upregulation of the MG catabolic process indicates an MG decrease, which leads to activation of the notch signaling pathway and could promote NPC proliferation. By analyzing the bulk-RNA-seq results, we noted that the mRNA expression level of the key MG catabolic factor *Glo1* was upregulated. RT-qPCR and western-blot analysis showed that the expression of Glo1 was obviously increased in cKI^f/+^ mice (Fig. 7c and Supplementary Fig. S7a, b). Since SERPINA3 is expressed in the nucleus, it could function as a transcriptional factor or co-factor.

Then, we examined whether *SERPINA3* binds to the *Glo1* promoter region using a chromatin immunoprecipitation-qPCR (ChIP-qPCR) experiment. We designed eight pairs of primers targeting different regions of the *Glo1* gene (from −5 kb to the negative control coding sequence; Supplementary Table S2) for enrichment analysis (Fig. 7d). The results indicated that human SERPINA3 was enriched in a region of the *Glo1* promoter surrounding position 2.5 kb−1.5 kb from the transcriptional start site. Next, to demonstrate the transcriptional regulatory function of SERPINA3, we cloned the *Glo1* promoter region from 0 to −3 kb into the psiCheck2 vector and performed a luciferase assay. The results showed that SERPINA3 increased the expression of luciferase by binding with the *Glo1* promoter (Supplementary Fig. S7c).

To further validate that *Glo1* is downstream of SERPINA3, we performed a knockdown experiment through the IUE of a specific short hairpin RNA(Glo1-shRNA) to suppress Glo1 expression. Here, we observed that the distribution of GFP^+^ cells in the VZ/SVZ of the rescue group (SERPINA3 + Glo1-shRNA) was reduced to the level of that in the control group (Fig. 7e, f). In addition, the increase in the proportion of SOX2^+^GFP^+^ and SOX2^+^TBR2^−^GFP^+^ cells in the expanded SVZ, caused by SERPINA3 overexpression, was restored by downstream Glo1 inhibition at E15.5 (Fig. 7g–i). The knockdown experiments provide further evidence that SERPINA3 regulates the proliferation of NPCs through Glo1.

To determine whether the overexpression of Glo1 can mimic the effects of SERPINA3 overexpression, Glo1 was overexpressed in the E13.5 mouse neocortex through IUE. We observed that the distribution of GFP^+^ cells was altered with an increase in the proportion of GFP^+^ cells in the VZ/SVZ and IZ, and there was a decrease in the CP (Supplementary Fig. S7d, e). EdU^+^GFP^+^ cells were increased in the VZ/SVZ region at E15.5 compared to the level in the control group (Fig. 7j, k). This effect of Glo1 overexpression was also confirmed by an increase in the proportion of cells stained with SOX2 (Supplementary Fig. S7f, g), suggesting that Glo1 could promote progenitor proliferation, similar to SERPINA3. We also found that approximately one-third of mice electroporated with the *Glo1* overexpression vector at E13.5 showed a slightly folding-like structure in the cortex at regions containing electroporated cells in P0 mice (Fig. 7l). In addition, we observed that the overexpression of Glo1 increased the percentage of SATB2^+^GFP^+^ neurons (Supplementary Fig. S7h, i).

## Discussion

During evolution, the emergence of cortical folding and gyrification has enabled humans and many higher mammals to cope with and process more complex living issues. Several folding mechanisms have been reported. Among them, increased number of neurons and external constraints are strongly related to gyrification and also well-known^35,36^. The curved cortex surface has a higher capability of containing billions of neurons in a limited cranial space, which allows humans and other higher mammals to have more functional neurons, which can form large-scale neural circuits, and further obtain higher cognitive functions^12,32^. Recent studies have explored the association between the relative abundance of oRG and the degree of cortical folding in various species. Some human genes also have been for their functions in this process, such as *ARHGAP11B*, *TMEM14B*, and *TBC1D3*. Notably, they were found to promote cortex folding through similar cellular mechanisms. The increase in neural progenitor proliferation and neuronal output induced by the overexpression of these genes through different pathways eventually contributes to this folding^13,16,37^.

**Fig. 8:**
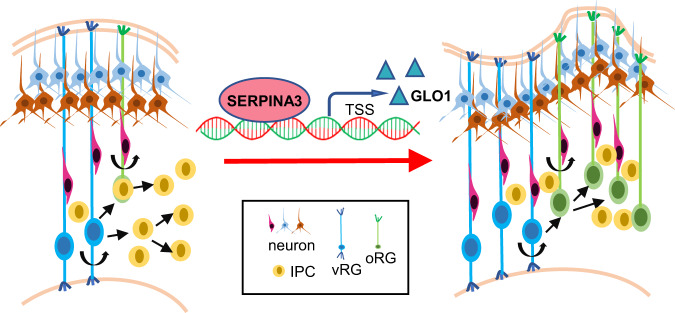
Proposed model for the role of SERPINA3 in cortical expansion and folding. SERPINA3 targets *Glo1* to increase the expansion of NPCs, especially BPs, giving rise to an increased number of neurons and leading to cortical folding.

Here, we observed a remarkable effect of the human *SERPINA3* gene in promoting cortex expansion and folding. Our data reveal an important mechanism by which SERPINA3 targets *Glo1*, promoting the proliferation of NPCs. SERPINA3 increases the expansion of NPCs, especially BP cells, giving rise to an increased number of neurons and leading to cortical folding (Fig. 8). In other studies, SERPINA3 was found to increase the telomere length in liver hepatocytes, and the downregulation of SERPINA3 expression in bone marrow inhibits the retention of hematopoietic progenitor cells and promotes their differentiation and migration^23,38^. These results indicate that SERPINA3 is related to cell proliferation. In E15 cKI^f/+^ mice, levels of NPC proliferation markers SOX2, PAX6, and PH3 were significantly increased. Expression of the oRG marker HOPX was also increased^13,39^. Enhanced NPC proliferation and the generation of oRG lead to an enlarged neural progenitor pool, providing a larger and longer neuronal output, leading to cortical folding, which fits the neuron number-related cortical folding model reported by others^11–13^.

It is unclear whether there are specific types or layers of neurons that increase and induce cortical folding. Alternatively, it is possible that all neuron types increase at the same ratio in cKI^f/+^ mice and induce folding. We analyzed the ratio of different neuron types in the P0 cKI^f/+^ mouse cortex by sc-RNA Seq. Interestingly, the ratio of layer II/III neurons to total neurons increased, whereas the percentage of deep-layer neurons decreased. However, the number of deep-layer neurons was not decreased based on our immunostaining results. Combined with immunostaining, we found that total neurons were increased, and the increase in the number of upper-layer neurons was greater, which decreased the ratio of deep-layer neurons. The increased upper-layer neuron pattern is similar to the phenotype in the monkey cortex reported recently^37^. As reported in primates, the increased proliferative capacity of oRG results in an increase in BP numbers and a prolonged phase of late-born neuron production, which will ultimately be upper-layer neurons^7,40,41^.

Some studies have shown that upper-layer neurons, especially layer II/III neurons, might have crucial functions in cognition^14,42^. Here, we also confirmed the improvement in working memory and learning ability in cKI^f/+^ mice by using the Morris water maze and radial arm maze behavior tests. Although further neural circuit analyses are required to evaluate the behavioral consequences of SERPINA3-induced cortical expansion and gyrification, the increased memory ability observed in the cKI^f/+^ mice is in line with the expanded neocortex and increased abundance of upper-layer neurons in these mice. However, several questions still need to be further explored. Here we did not provide direct evidence to show that the improvement in cognition resulted from gyrification. We also do not know the exact neural circuit changes that occurred in our mouse model. Moreover, whether there are more functional neural circuits formed by the increased number of neurons or whether the number of neural circuits remains constant while the size of the neural circuits is increased are unknown. It is important to note that SERPINA3 overexpression was driven by the CAG promoter, and the expression level might be higher than that under physiological conditions, whether SERPINA3 induces folding at that level needs to be further studied.

Through bulk-RNA-seq analysis of the cKI^f/+^ mouse cortex, the downstream target gene *Glo1* was identified. The overexpression of Glo1 enhanced the proliferation of NPCs, which is consistent with the functions of Glo1 reported by other groups^24,43^. The long-term overexpression of Glo1 in neural stem cells by IUE also induced cortical folding.

We also considered whether the SERPINA3-Glo1 pathway could also be involved in human cortical folding. As reported in other papers and through the SAFRI autism candidate gene database, Glo1 mutations are highly related to autism, a neurodevelopmental disorder^44,45^. Autism is often accompanied by developmental delays, increasing our suspicion that Glo1is related to human neurogenesis. Knocking out Glo1 in hiPSC-derived neurons leads to a decrease in cell viability and an increase in caspase-3 activity^43^. This in vitro study strengthens our confidence that the SERPINA3-Glo1 pathway regulates neurogenesis in the human brain and might be one of the candidate mechanisms related to human cortex folding. Meanwhile, we speculate that more research is needed to be done to validate the possible function of SERPINA3 in human neurogenesis and cortex folding.

In summary, we found that the human *SERPINA3* gene promotes neurogenesis, cortical expansion, and folding by modulating the neurodevelopment-related *Glo1* gene. Our work could provide a better understanding of human cortical folding and cognitive development.

## Materials and methods

### Human samples collection

The collection of human aborted embryos was performed at Peking University Third Hospital. This research was approved by the Reproductive Study Ethics Committee of Peking University Third Hospital and the institutional review board (ethics committee) of the Institute of Zoology (NO. IRB00006761-M2020205). Informed consent was provided to and signed by donor patients prior to collection of fetal tissue samples. All protocols followed the Interim Measures for the Administration of Human Genetic Resources, administered by the Ministry of Science and Technology of China.

### Animals

ICR mice and C57BL/6 mice were purchased from Beijing Vital River Laboratory Animal Technology Co. Ltd. And they were housed in the institutional animal care facility with a 12-h light to dark schedule. During the whole project, all mice used in this study were taken care according to the Guide for the Care and Use of Laboratory Animals. And all animal experiments were approved by the Animal Committee of the Institute of Zoology, Chinese Academy of Sciences.

### Knock-in mice construction

hSERPINA3^flox/+^ mice were generated using gene editing technology based on CRISPR/Cas9 technology. In brief, target fragment CAG-loxP-STOP-loxP-3×flag-hSERPINA3-WPRE-PA was inserted into Rosa26 site between exon1 and exon2. Cas9 vector and Donor vector cloned with CAG-loxP-STOP-loxP-3×flag-hSERPINA3-WPRE-PA flanked with 5′homologous arm/3′homologous arm was microinjected into zygotes. 224 zygotes were implanted into surrogate mother mice. After genotyping of newborn pups, two of them were identified as SERPINA3^flox/+^ mouse (F0). F0 mice were breed with B6D2F2 mice. The offspring (F1) mice were confirmed with genotyping PCR products sequencing. Then F1 mice were crossed with the Nestin-Cre mice (C57BL/6 background) to generate hSERPINA3^flox/+^; Nestin-Cre mice.

### Plasmid construction

*SERPINA3* and *Glo1* cDNAs were acquired by reverse transcription of human and mice brain total RNA and subcloned into the PCDH-CAG-GFP-FLAG vector to generate the FLAG-tagged pCDH-*SERPINA3* and pCDH-*Glo1* plasmid. ShRNAs for the target genes were cloned into the pSicoR-GFP vector.

The sequences for shRNAs were as follows: SERPINA3-shRNA,5′-CCGGAGTCTCCCAGGTGGTCCATAACTCGAGTTATGGACCACCTGGGAGACTTTTTTG-3′. Glo1-shRNA,5′-TCTCGCTCTACTTCTTAGCTTACTCGAGTAAGCTAAGAAGTAGAGCGAGTTTTTG-3′.

### Cell culture

HEK 293FT and Neuro 2a cell (N2A) lines were cultured in DMEM medium with 10% FBS, non-essential amino acid, and penicillin/streptomycin. H9 human ES cells were cultured in Essential 8 medium (Thermo, A1517001) and maintained on Matrigel-coated six-well plates (Corning, 354277).

### Human NPC induction

Human NPC induction was based on previous study^46^. In brief, when H9 ES cells reached 70%–80% in six-well plate, medium was replaced with NPCs induction medium including 50% (v/V) DMEM/F12 (Invitrogen), 50% (v/V) neurobasal medium (Invitrogen), 1× B27 supplement (Invitrogen), 1× N2 supplement (Invitrogen), 1× GlutaMAX, 2 × 10^−6^ M dorsomorphin (Sigma), 10 ng mL^−1^ hLIF (Millipore), 4 × 10^−6^ M SB431542 (Sigma), 4 × 10^−6^ M CHIR99021 (Sigma), and 0.2 × 10^−6^ M compound E (EMD Chemicals). Cells were cultured for 7 d and the medium was changed every other day. Then, cells were separated into single cell by Accutase and transferred to six-well (100 000/well) or 24-well (8000/well) Matrigel coated plates, and maintained in human neural progenitor medium (50% (v/v) neurobasal medium, 50% (v/v) DMEM/F12, 1× B27, 1× N2 supplement, 1× GlutaMAX, 10 ng mL^−1^ hLIF, 3 × 10^−6^ M CHIR99021, and 3 × 10^−6^ M SB431542). For neuronal differentiation, human NPCs were cultured for 3 d and then cells were incubated in neuronal induction medium (DMEM/F12 medium containing 1× B27 supplement, 1× N2 supplement, 400 × 10^−6^ M dbcAMP (Sigma), 10 ng mL^−1^ BDNF (Peprotech), and 10 ng mL^−1^ GDNF (Peprotech)). Laminin (Sigma, 1 µg mL^−1^) was added after 2 d culture. Cells were cultured and changed the medium every other day.

### Generation of cerebral organoids

Generation of cortical organoids from H9 human ES cell (hESC) lines was conducted as previous study^47^. Briefly, hESCs were dissociated to single cell using Accutase for 6 min at 37 °C. Cells were collected in E8 medium with ROCK inhibitor (50 × 10^−6^ M) and about 10,000 cells were plated in each well of low-attachment 96-well plate (Sumitomo Bakelite) to form EBs. The next day, the medium was changed completely with low-bFGF hESC medium (80% (v/v) DMEM-F12, 8.5% (v/v) KOSR, 1.5% (v/v) ESC-quality FBS, 1× GlutaMAX, 1× MEM-NEAA, and 4 ng mL^−1^ bFGF). The EBs were fed every other day without disturbing the EB, for 5 days. Then, each EB was transferred to low-attachment 24-well plate containing neural induction medium (DMEM-F12 with 1× N2 supplement, 1× GlutaMAX, 1× MEM-NEAA, and 1 µg mL^−1^ heparin). After 5 days in neural induction medium, the aggregates were transferred one by one to each Matrigel droplet and cultured in 10 mm dish with cerebral differentiation medium (50% (v/v) neurobasal medium, 50% (v/v) DMEM/F12, 1× B27, 1× N2 supplement, 1× GlutaMAX, and 0.02% (v/v) human insulin) without vitamin A. After 4 days in static culture, the embedded organoids were transferred to a 100 mL^−1^ spinning bioreactor and cultured in 70–85 mL cerebral differentiation medium containing vitamin A. The medium was changed completely every week.

### GI measurement

The method we use was to calculate the ratio between the lengths of the total to that of the superficially exposed cortical surface as previously described^28–30^. GI = Length of complete contour/Length of outer contour.

### Lentivirus production and infection

The detailed procedure for lentivirus preparation was described previously^48^. Lentiviral package plasmids and the core plasmids were transfected into HEK 293FT by GenEscort I (Wisegen). Supernatants containing virus were collected at 24, 48, and 72 h after transfection and centrifuged at 3000 rpm for 5 min to remove cell debris. For lentivirus infection, MOI (multiplicity of infection) was set to 5, and the incubation time was 12 h. 2 µg/mL polybrene was added to infection medium to improve the efficiency of infection. After infection, cells were cultured continually and used for further analysis.

### Western blotting

Cells and tissue were lysed with Ultrasonic Homogenizer in RIPA buffer (10 mM PMSF and proteinase inhibitor mix). After being centrifuged and mixed with loading buffer, the protein samples were loaded onto SDS-PAGE gel for electrophoresis, and the bands were transferred to nitrocellulose or polyvinylidene fluoride membranes. The membranes were blocked with 5% nonfat milk in PBST buffer (PBS with 0.05% Tween-20) for 1 h at room temperature, and incubated with primary antibody at 4 °C overnight. Donkey anti Rabbit/mouse/goat IgG secondary antibodies (LI-COR Bioscience) were used to visualize the bands with Odyssey Infrared Imaging System.

### IUE

IUE was performed according to the previously reported protocol^49^. In brief, a timed pregnant mouse at E13.5 was anesthetized by given an injection of pentobarbital sodium. Then the uterine horns were gently exposed. Recombinant plasmid mixed with fast green (2 mg/mL; Sigma) was microinjected into the fetal brain ventricles with glass capillaries. And the mixed plasmids were electroporated into the neural stem cells through electroporator (Manual BTX ECM830) with 5 pulses of 36 V current with 50 ms duration and a 950 ms interval. After electroporation, the pregnant mice were sacrificed at different time point for phenotype analysis. The fetal brains were fixed in 4% paraformaldehyde (PFA) overnight, then dehydrated in 30% sucrose at 4 °C and cut into sections for further analysis.

### RT-qPCR

The extraction of total RNA and reverse-transcription of complementary DNA were performed as previously described^50^. Quantitative real-time PCR (RT-qPCR) was performed in a 20 µL reaction mixture using Super Real SYBR Green PreMix Plus kit (TIANGEN) on ABI 7500 real-time PCR system (Applied Biosystems). The primers used for RT-qPCR were described in Supplementary Table S2.

### BrdU/EdU labeling

For BrdU labeling, the pregnant mice were injected with 50 mg/kg BrdU 2 h before embryonic brains were harvested and fixed at E15. Adult mice were injected with BrdU (50 mg/kg) seven times with a 2 h interval in-between. They were sacrificed 24 h after the last injection.

For EdU labeling, the pregnant mice were injected with 50 mg/kg EdU 2 h before embryonic brains were harvested and fixed at E15.

### Immunostaining

The procedure for immunostaining brain sections or cultured cells was described previously^51^. Immunostaining for cultured cells or brain slices was performed according to the following procedure: the samples were washed with PBST (1% Triton X-100 in 1 M PBS), fixed in 4% PFA, rewashed for 3 times with PBST, then blocked by 5% BSA (in 1% PBST) for 1 h, incubated with primary antibodies overnight at 4 °C, and visualized with fluorescence-labeled secondary antibodies. DAPI solution (2 μg/μL) was used for nuclear staining. After sealing the slides with 50% glycerin, these slices were scanned on Zeiss LSM 880 confocal microscope and analyzed with Zeiss Zen software. The antibodies used were described in Supplementary Table S3.

### ChIP–qPCR

ChIP analysis was performed essentially as previously described^52^. Briefly, after transfected with flag-tagged SERPINA3 vector 48 h, N2A cells were crosslinked with 1% fresh formaldehyde solution (50 mM, HEPES-KOH, pH 7.5, 100 mM NaCl, 1 mM EDTA, 0.5 mM EGTA and 1% formaldehyde) for 10 min at room temperature, and the reaction was quenched with 2.5 M glycine. After washing twice with ice-cold PBS, cells were resuspended in lysis buffer (50 mM HEPES-KOH, pH 7.5, 140 mM NaCl, 1 mM EDTA, 10% glycerol, 0.5% NP-40, 0.25% Triton X-100, 1× protease inhibitors) for 10 min at 4 °C. Nuclei were isolated at 4000 rpm for 5 min at 4 °C and resuspended in lysis buffer 2 (10 mM Tris-HCl, pH 8.0, 200 mM NaCl, 1 mM EDTA, 0.5 mM EGTA, 1× protease inhibitors) and then rocked gently at room temperature for 10 min. Nuclei were sonicated in lysis buffer 3 (1% SDS, 10 mM EDTA, 50 mM Tris-HCl pH 8.1, 1× Protease Inhibitor) with Scientz-IID sonicator. Chromatin was immunoprecipitated overnight at 4 °C with 50 µL Protein A beads (Dynabeads, Invitrogen) pre-coated with 2 µg FLAG or anti-goat IgG antibodies. Beads were washed three times with low-salt buffer (0.1% SDS, 1% Triton X-100, 2 mM EDTA, 150 mM NaCl, Tris-HCl, pH 8.1) and three times with high-salt buffer (0.1% SDS, 1% Triton X -100, 2 mM EDTA, 500 mM NaCl, Tris-HCl, pH 8.1) at room temperature. Immunocomplexes were eluted by using TES buffer (50 mM Tris-HCl, pH 8.0, 10 mM EDTA and 1% SDS) overnight at 65 °C. After extracted by TIANamp Genomic DNA Kit (Tiangen), DNA was used for RT-qPCR analysis. The primers used for RT-qPCR were described in Supplementary Table S2.

### Luciferase assay

The *Glo1* promoter was cloned into psiCHECK-2 vector for luciferase activity assay through dual-luciferase reporter assay system. SERPINA3 and control plasmid together with *Glo1* promoter luciferase plasmid at a ratio of 10:1 was transfected into N2A cells. Cells were collected to detect the expression of luciferase with the Promega kit by Promega Glomax after 48 h transfection.

### RNA-sequencing analysis

Total RNA from E15 telencephalic tissue of WT and cKI^f/+^ mice was extracted through TRIzol methods. After passed a quality control and quantification, total RNA was used for reverse transcription and library construction. RNA-sequencing analysis was performed on the Illumina HiSeq 2500 platform in Annoroad Genomics.

### Single cell samples preparation and transcriptome library construction

Artificial cerebrospinal fluid (ACSF) was prepared as previously described^53^. ACSF (including 87 mM NaCl, 75 mM sucrose, 26 mM NaHCO_3_, 20 mM glucoses, 7 mM MgSO_4_, 2.5 mM KCl, 1.25 mM NaH_2_PO_4_ and 1 mM CaCl_2_, pH = 7.4) was ice cooled and oxygenated before use. Cerebral cortex was collected from P0 WT and cKI^f/+^ mice and digested in papain for 30 min at 37 °C. The cell suspensions were filtered through 40 μm cell strainer and centrifuged at 200× *g* for 5 min, the supernatant was then discarded. The cell precipitation was resuspended by cold ACSF and washed twice. After washing, precipitated cells were resuspended with 1 mL 1× PBS containing containing 1% BSA to obtain single-cell suspension. The single-cell suspension was then processed with 10× Chromium Next GEM Single Cell 3′ GEM, Library & Gel Bead Kit v3.1 following the standard user guide. After quality control, the final transcriptome library was sequenced on Illumina HiSeq platform in Annoroad Genomics.

### Clustering and annotation

Sequencing and preprocessing through Cell Ranger were performed by Annoroad Genomics company. The barcode and matrix files generated by Cell Ranger were analyzed on R using Seurat package. Two datasets of WT and cKI^f/+^ were integrated and modulated by Harmony package. Then clusters were calculated at a resolution of 0.3. And all marker genes for each cluster were calculated with 0.25 min.pct and 0.25 log2fc.threshold. After obtaining 26 clusters and their marker genes, these clusters were annotated based on their top marker genes on published database form Allen brain institute, DropVis and mousebrain.org.

### Behavioral experiment

#### Open field test

The open-field test was used to analyze the locomotor activity. Mice (8–9 weeks old) were gently placed into an open field arena (40 cm width × 40 cm length × 40 cm height) and allowed to explore freely for 5 min. The locomotor activity and time spent in the center and margin areas were monitored and analyzed by the EthoVision XT v14 software. Testing occurred in a dimly lit room maintained at 30–40 lux.

#### Elevated-plus maze

The test was performed as previously described^54^. The elevated-plus maze has two open arms and two closed arms (each arm 40 cm × 9.5 cm; height of closed arms 9.5 cm). The maze stood 70 cm above the ground. Mice were placed in the center of the maze and allowed to explore freely for 5 min. The percentage of time that mice spent in the open and closed arms was analyzed.

#### Y-maze

Spontaneous alternation testing was carried out in the Y-maze test as previously described^55,56^. The procedure consisted of two phases: the training phase and the testing phase. During the training phase, mice were allowed to explore two arms of the Y-maze with the third arm (the ‘novel arm’) blocked for 5 min. After a 15-min interval, the testing phase was performed, and mice were allowed to explore all three arms freely for 5 min. Frequency and time for each arm entry were recorded by the EthoVision XT v14 software.

#### Novel object recognition test

The procedure was performed as previously described with some modification^57,58^. Briefly, the apparatus was a 40 cm × 40 cm × 40 cm box. Mice were placed to an empty area to habituate for 10 min. After 2 h, two identical objects (orange colored cuboid) were placed in the testing box. Mice were placed into the box and freely explored for 10 min. After 24 h, one object was randomly replaced with a novel object, a red colored cylinder. Mice were returned into the box to freely explore for 10 min. Object exploration was defined as percentage of novel object preference was calculated as: novel object exploration time/(novel object exploration time + familiar object exploration time) × 100%.

#### Morris water maze

Morris water maze procedure was performed as previously described^59^. It contained a circular tank and an escape platform. Water temperature was maintained at 20–22 °C during the entire training and testing session. Platform was submerged 1 cm lower than the surface of water in the training stage. In brief, the learning trials included 4 trials per day over 7 consecutive days. Mice were placed in four different starting positions every day. Mice were allowed to explore freely for 1 min. If mice touch or climb platform, we stopped the timer and recorded the time. If mice did not find the platform within 1 min, they were guided to find the platform and allowed to rest on the platform for 15 s. For probe trial test, the platform was removed. Mice were placed in a novel start position (180° from the original platform position) in the maze and were allowed to move up to 1 min. Data were recorded by the EthoVision XT v14 software.

#### Delayed non-matching-to-place radial eight-arm maze (DNMP-RAM)

The eight-arm radial maze was composed of eight arms (30 cm × 5 cm × 15 cm) and a center zone. The behavior test was performed by the previously published method with some modification^57,60^. Briefly, mice were restricted to 85%–90% of body weight. Testing included two trials per day over 6 consecutive days. One trial was consisted of a sample phase and a choice phase. For the sample phase, start arms and sample arms were opened, and other arms were blocked by board. Cheese as bait was placed into sample arm. Mice were placed in the maze with facing the end of the start arm and allowed to find cheese within 3 min. Then, mice were returned the cages. For the choice phase, bait was placed into the end of opening test arm separated by one closed arm (separation 2) or three closed arms (separation 4) from the new sample arm. Within 3 min, mice entered the test arm as a correct choice, while entered the sample arm or start arm as an incorrect. In order to exclude the older reference memory, the maze was rotated 45° and cleaned with 75% ethanol in the end of each stage.

#### Statistical analysis

Immunostaining brain slices were imaged by Zeiss LSM 880 confocal microscope and images were analyzed by ZEN2010 (Lecia) and Photoshop (Adobe Systems). For each experiment with mice or cells, at least 3 biological replicates were performed. Biological replicates are defined as independent experiments in cells or the same experiment with different mouse embryos. For all experiments, statistical analysis was performed using GraphPad Prism 8.0 with unpaired *t*-test. All Results presented means ± SEM.

## Supplementary information

Supplementary information

Supplementary Table S1

## Data Availability

All data needed to evaluate the conclusions in the paper are present in the paper and/or in the Supplementary Materials. The Bulk-RNA sequencing and sc-RNA Seq data have been uploaded to NCBI’s GEO. All the data could be accessed through GEO accession numbers GSE165345 and GSE165440.
