# Supplementary material for: Human SERPINA3 induces neocortical folding and improves cognitive ability in mice
Source: Cell Discov. 2022 Nov 22;8:124. doi: 10.1038/s41421-022-00469-0 (PMC9681744; doi:10.1038/s41421-022-00469-0)
Supplement: Supplementary file 1 — Supplementary information [file 41421_2022_469_MOESM1_ESM.pdf]

Supplementary information

Human SERPINA3 induces neocortical folding and improves cognitive ability in mice

Jinyue Zhao, Chao Feng, Wenwen Wang, Libo Su, Jianwei Jiao

Inventory of supporting information

Supplementary Figures S1-S7

Supplementary Tables S1-S3

Supplementary Fig. 1

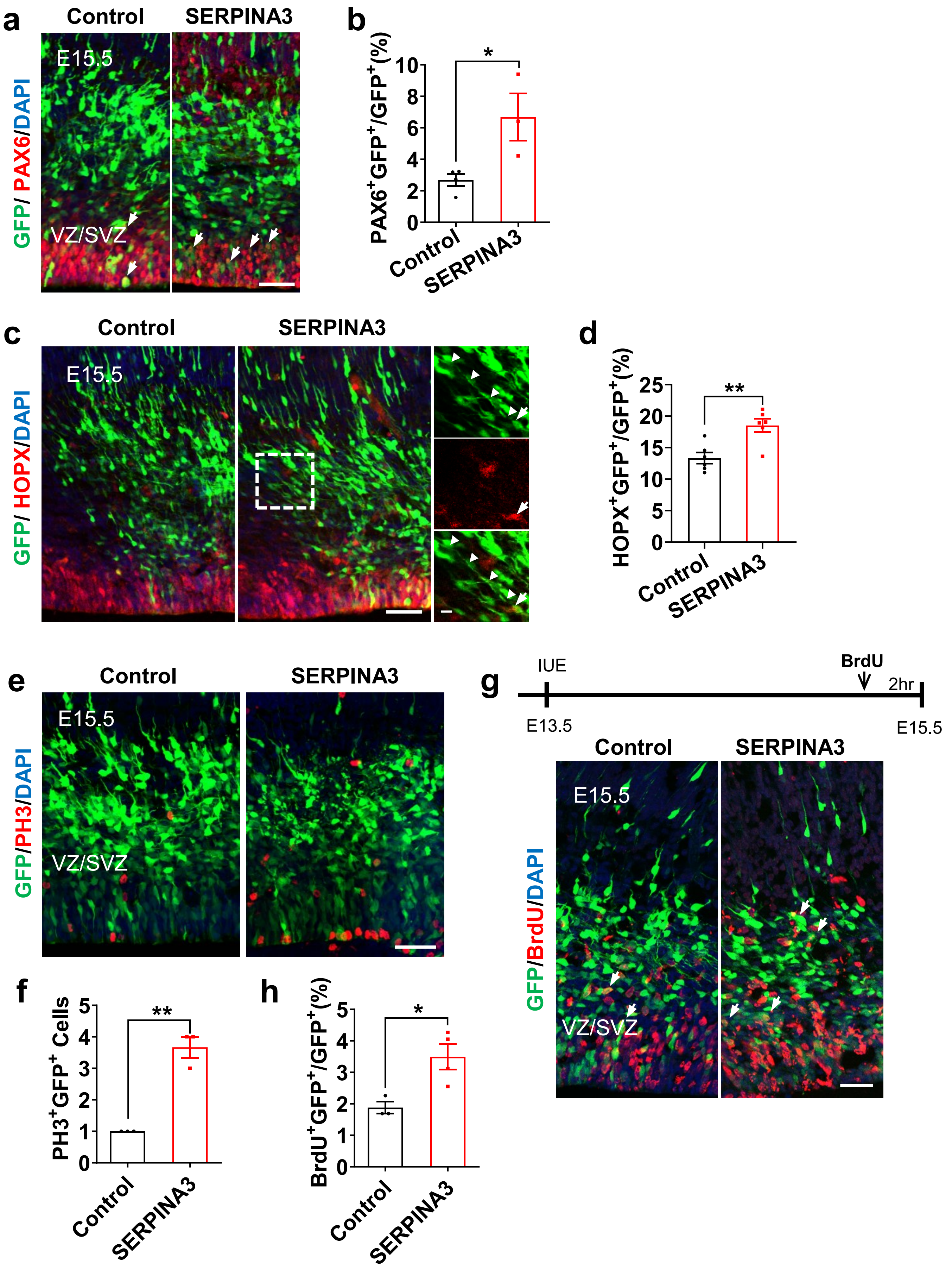

Supplementary Figure 1. Human SERPINA3 overexpression promotes NPCs proliferation in mice. a IF for PAX6 in E15.5 mice after IUE at E13.5. Arrows, $PAX6^+GFP^+$ cells. Scale bar, 50μm. **b** Quantification for the percentage of $PAX6^+$ cells among GFP^+ cells (control n=4, SERPINA3 n=3). **c** IF for HOPX in E15.5 mice after IUE at E13.5. Dashed white rectangle showed extra SVZ region. Arrows showed HOPX and GFP double positive cell with basal process. Scale bars, 50μm (left), 10μm (right). **d** Quantification for the percentage of $HOPX^+$ cells among GFP^+ cells (n=6). **e** IF for PH3 in E15.5 mice after IUE at E13.5. Scale bar, 50μm. **f** Quantification for the percentage of $PH3^+$ cells among GFP^+ cells (n=3). **g** IF for BrdU in E15.5 mice after IUE at E13.5. Axis shows the injection time of BrdU. Arrows, $BrdU^+GFP^+$ cells. Scale bar, 50μm. **h** Quantification for the percentage of $BrdU^+$ cells among GFP^+ cells (control n=3, SERPINA3 n=4). Two-tail unpaired T-test is used to analyze the data, n.s. (no significant difference), $P<0.05$ (*), $p<0.01$(**), $p<0.001$(***).

Supplementary Fig. 2

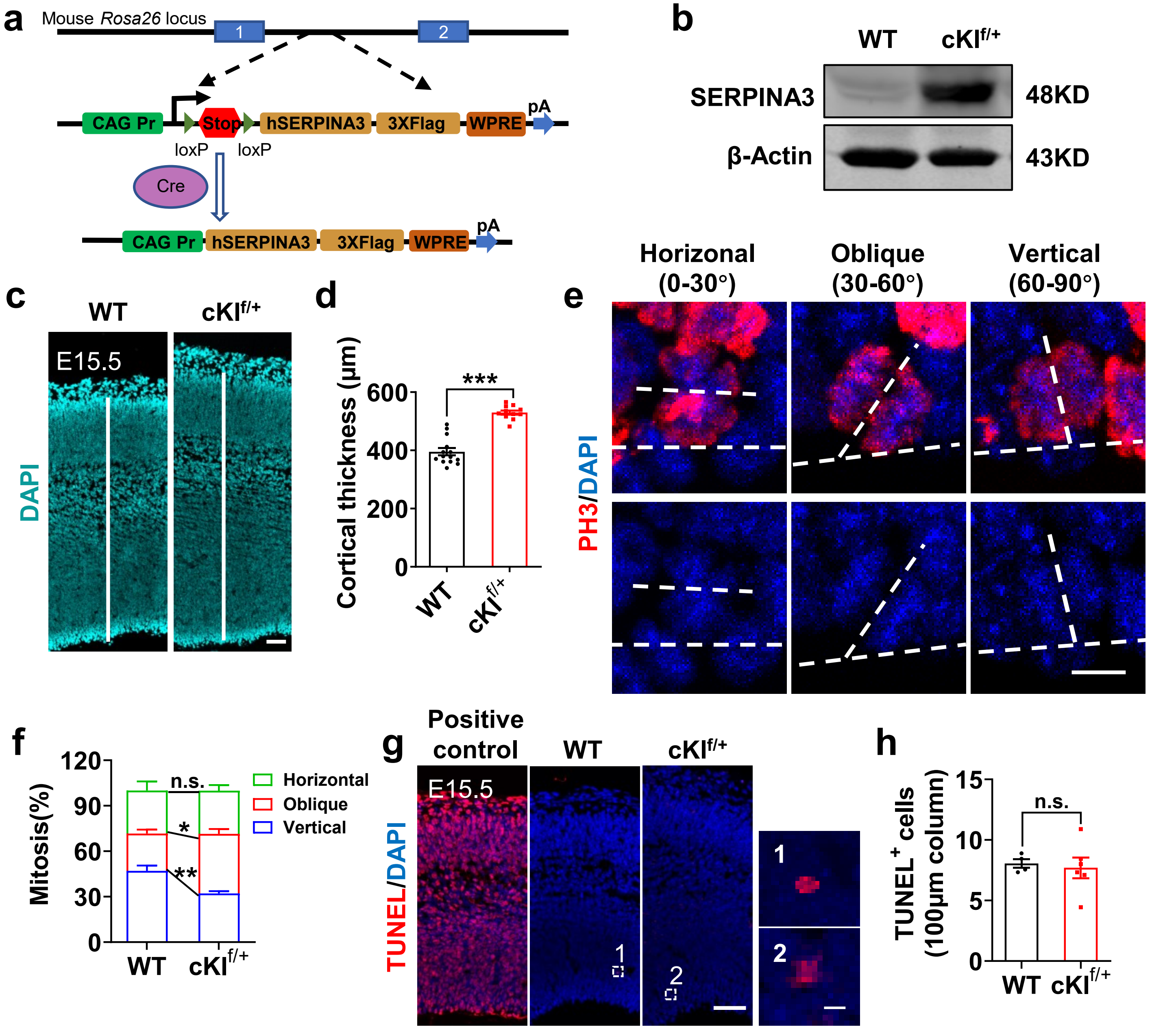

Supplementary Figure 2. Overexpression of human SERPINA3 promotes NPCs generation in mice. a Schematic strategy of generating $cKI^{f/+}$ mice. **b** Protein levels of SERPINA3 in cortex of WT and $cKI^{f/+}$ were measured by western blotting. β-actin was detected as a loading control. **c** Staining for DAPI in E15.5 WT and $cKI^{f/+}$ cortex. White lines indicate the cortical thickness. Scale bar, 50μm. **d** Quantification for the thickness of the cortical wall at E15.5 (WT n=14; $cKI^{f/+}$ n=11). **e** IF for PH3 in mitotic vRG cells of E15.5 mice. Different plane angle examples were showed. Horizonal 0-30°; Oblique 30-60°; Vertical 60-90°; Scale bar, 5μm. **f** Quantification for the plane of division at ventricle surface of vRG (WT n=3; $cKI^{f/+}$ n=6). **g** IF for TUNEL in WT and $cKI^{f/+}$ mice. Images 1 and 2 are high-magnification images. Scale bars, 50μm (left), 5μm (right). **h** Quantification for the $TUNEL^{+}$ cells (WT n=4; $cKI^{f/+}$ n=6). Two-tail unpaired T-test is used to analyze the data, n.s. (no significant difference), $P<0.05$ (*), $p<0.01$(**), $p<0.001$(***).

Supplementary Fig. 3

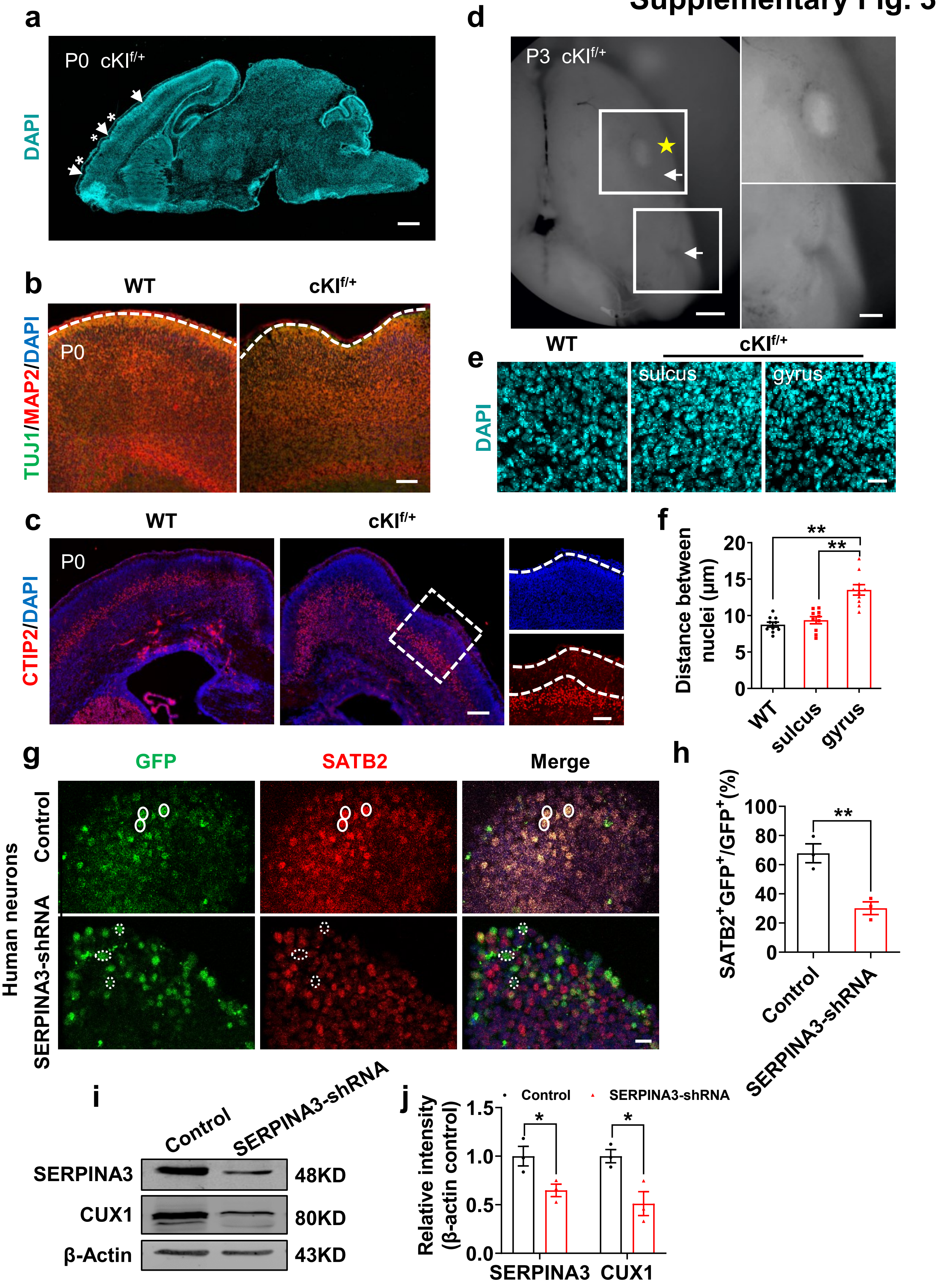

Supplementary Figure 3. Overexpression of human SERPINA3 leads to gyrification in mice. a Staining for DAPI in P0 $cKI^{f/+}$ cortex. Asterisks and arrows indicate gyrus and sulcus structure. Scale bar, 500μm. **b** IF of TUJ1 and MAP2 in P0 WT and $cKI^{f/+}$ cortex. Scale bar, 50μm. **c** IF of CTIP2 in P0 WT and $cKI^{f/+}$ cortex. Dashed white rectangle showed folding cortex. Scale bars, 200μm (left), 100μm (right). **d** A bright field of P3 $cKI^{f/+}$ brain. Yellow asterisk showed gyrus, arrow showed sulcus. Scale bars, 500μm (left), 200μm (right) **e** Staining of nuclei with DAPI in WT and $cKI^{f/+}$ cortex. Scale bar, 20μm. **f** Quantification of nuclei distance in different structure of $cKI^{f/+}$ cortex and WT (n=10). **g** IF for SATB2 in *SERPINA3*-shRNA lentivirus-infected human neurons. Outlined by white lines, $GFP^{+}SATB2^{+}$cells. Outlined by dashed white lines, $GFP^{+}SATB2^{-}$ cells. Scale bar, 20μm. **h** Quantification for the percentage of $SATB2^{+}$ cells among GFP^{+} cells (n=3). **i** Protein levels of SERPINA3 and CUX1 in *SERPINA3*-shRNA lentivirus-infected human NPCs and neurons were measured by western blotting. β-actin was detected as a loading control. **j** Statistics of relative band intensity of SERPINA3 and CUX1 (n=3). Two-tail unpaired T-test is used to analyze the data, n.s. (no significant difference), $P<0.05$ (*), $p<0.01$(**), $p<0.001$(***).

Supplementary Fig. 4

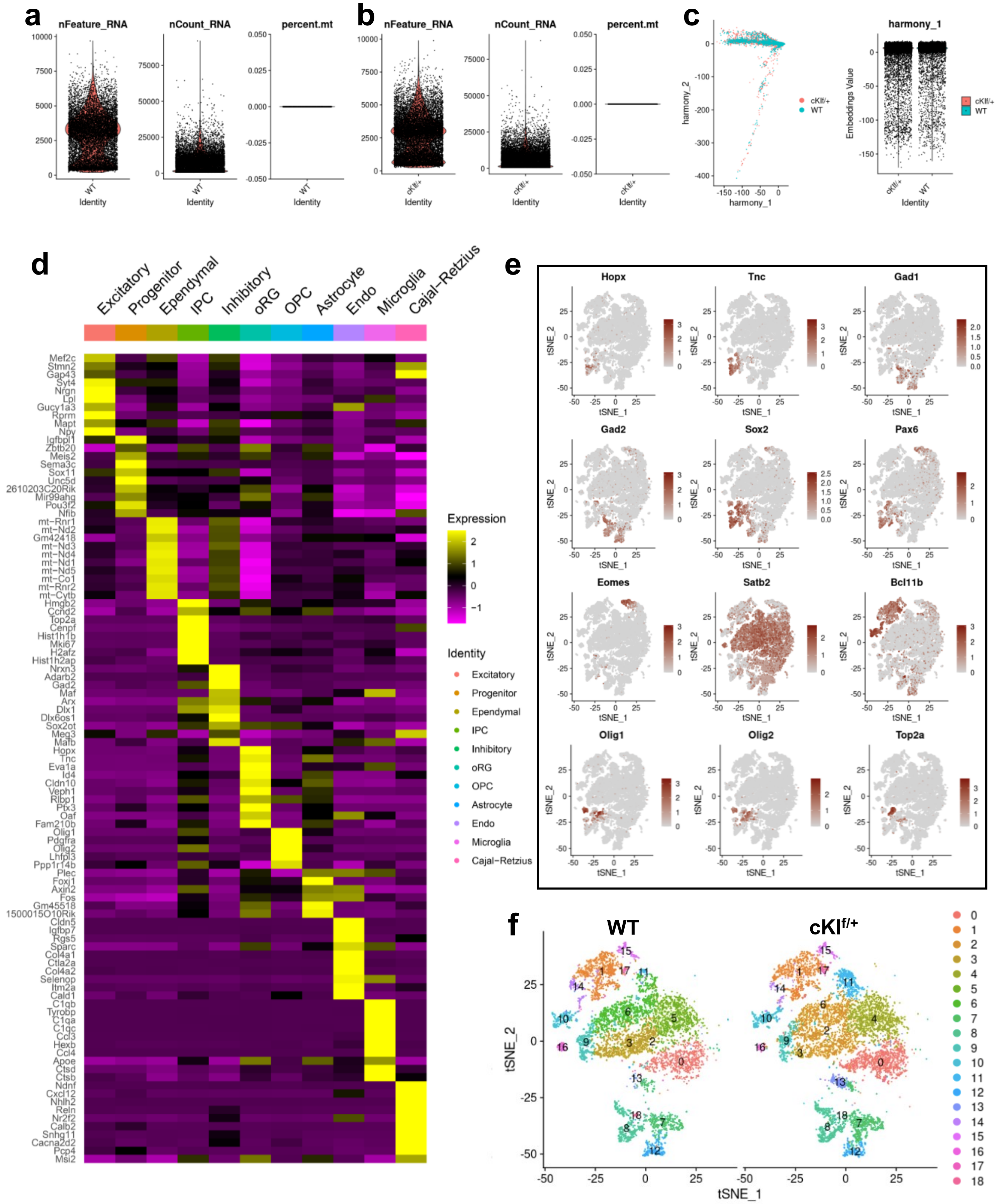

Supplementary Figure 4. ScRNA sequencing data pre-processing and top markers for each cell type. **a** Violin plots show the quality control of WT sequencing data. **b** Violin plots show the quality control of cKI$^{f/+}$ sequencing data. **c** Integration of WT and cKI$^{f/+}$ sequencing data. **d** Heatmap of marker genes of each cell type. Gene names were showed in the left, detailed cell type information was in the right part. **e** Feature plots for marker genes of each cluster. **f** Unsupervised clustering of neuron types in TSNE plot.

Supplementary Fig. 5

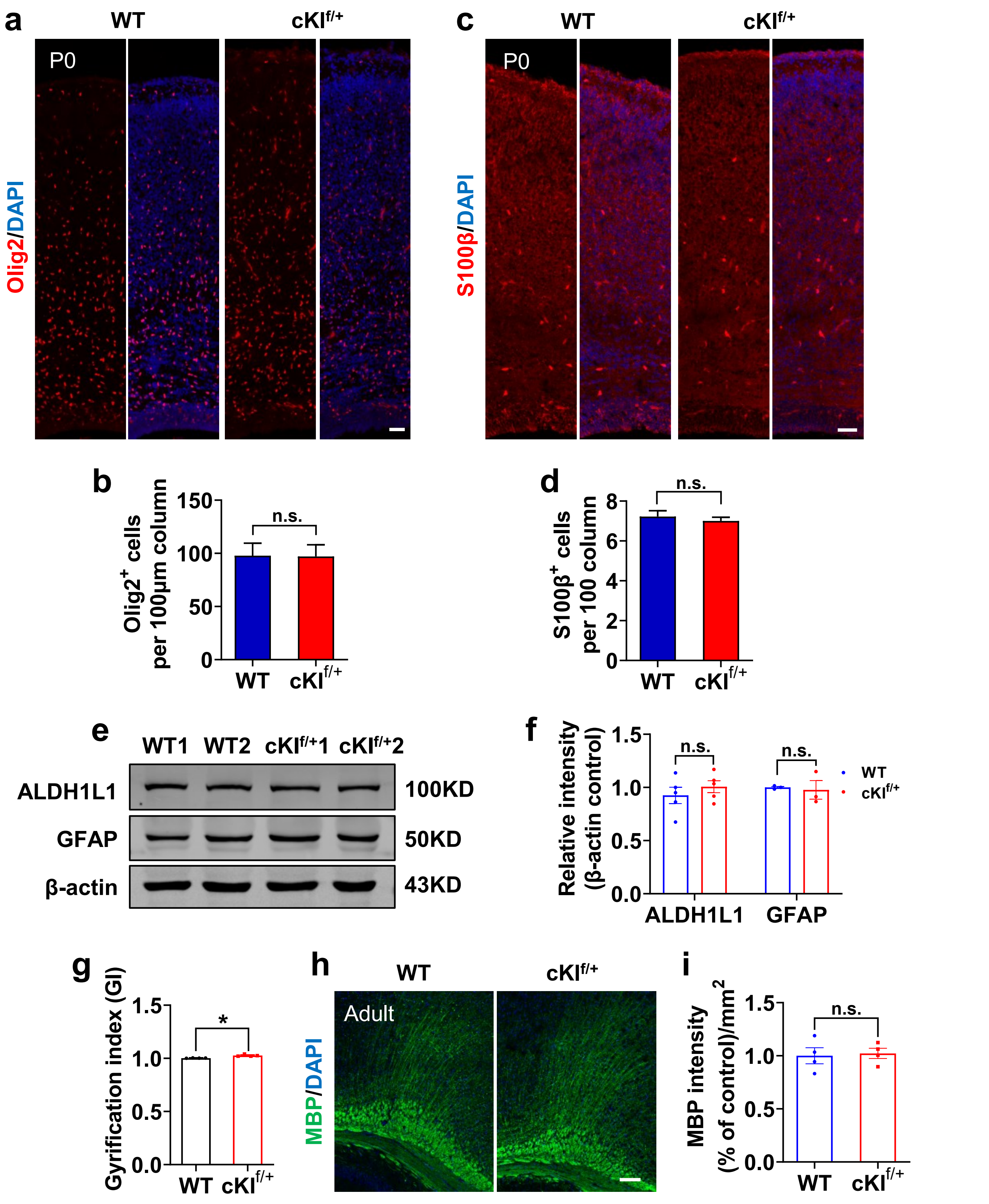

Supplementary Figure 5. Overexpression of SERPINA3 has no impact on gliogenesis. **a** IF for Olig2 combined with DAPI staining of P0 WT and cKI$^{f/+}$ mouse neocortex. Scale bar, 50 μm. **b** Quantification for Olig2^{+} cells in a 100 μm-wide column of P0 WT and cKI$^{f/+}$ mouse neocortex (n=3). **c** IF for S100β combined with DAPI staining of P0 WT and cKI$^{f/+}$ mouse neocortex. Scale bar, 50 μm. **d** Quantification for S100β^{+} cells in a 100 μm-wide column of P0 WT and cKI$^{f/+}$ mouse neocortex (n=3). **e** Protein levels of ALDH1L1 and GFAP in cortex of P0 WT and cKI$^{f/+}$ were measured by western blotting. β-actin was detected as a loading control. **f** Quantification for the protein level of ALDH1L1 and GFAP in WT and cKI$^{f/+}$ cortex (n=5 and 3). **g** Quantification for the GI of the adult cortex (n=4). **h** IF for MBP combined with DAPI staining of adult WT and cKI$^{f/+}$ mouse neocortex. Scale bar, 100 μm. **i** Quantification for MBP^{+} intensity of adult WT and cKI$^{f/+}$ mouse neocortex (n=4). Two-tail unpaired T-test is used to analyze the data, n.s. (no significant difference), $P<0.05$ (*), $p<0.01$(**), $p<0.001$(***).

Supplementary Fig. 6

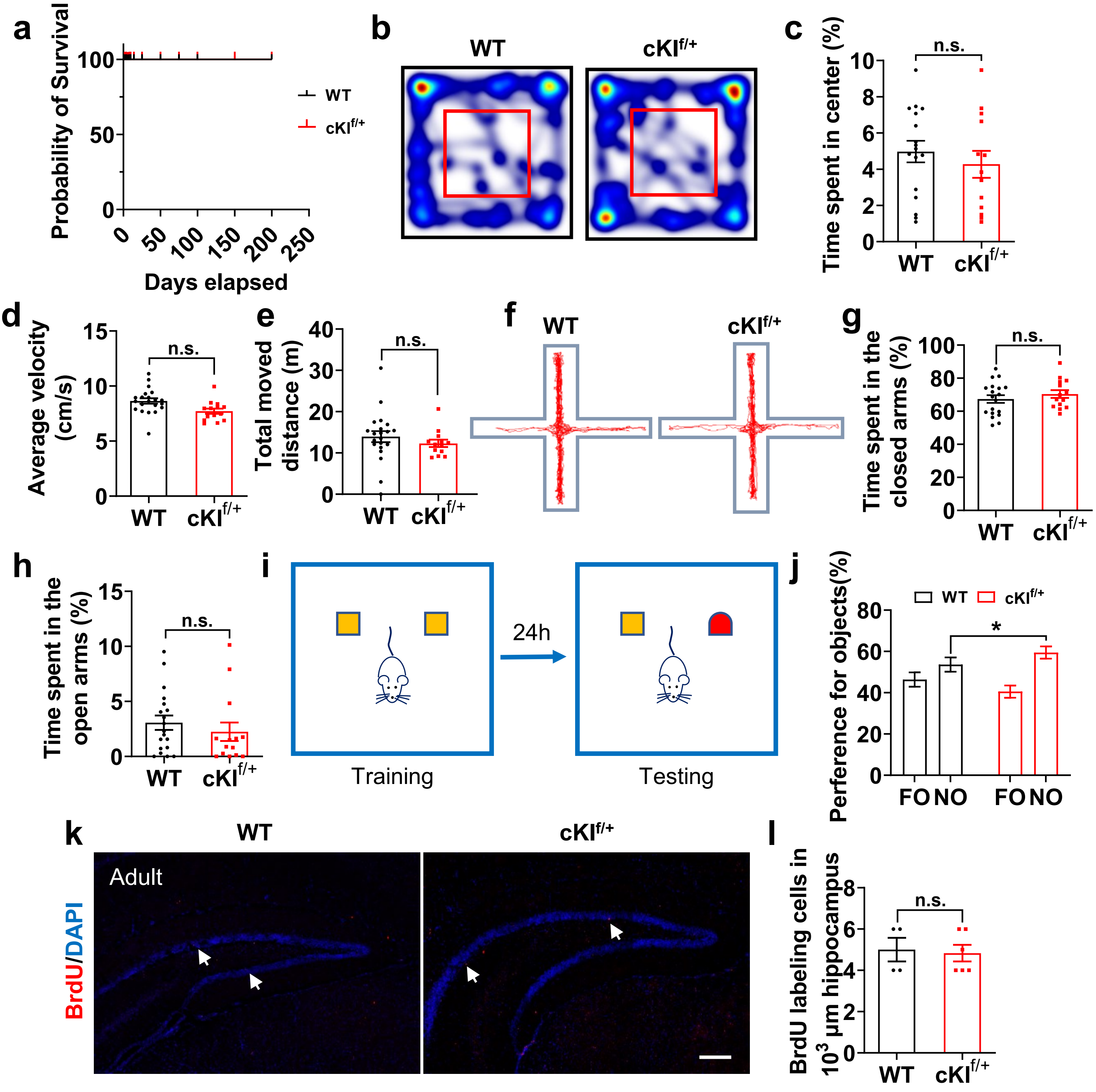

Supplementary Figure 6. Human SERPINA3 does not lead to abnormal behaviors in mice. a The survival curve of WT and $cKI^{f/+}$ mice. **b** Representative tracks of WT and $cKI^{f/+}$ mice in the open field test. The red box represents the center area. **c** Quantification for the percentage of time spent in the center between WT and $cKI^{f/+}$ mice in the open field test (WT n=17; $cKI^{f/+}$ n=13). **d** Quantification for the average velocity between WT and $cKI^{f/+}$ mice in the open field test (WT n=21; $cKI^{f/+}$ n=14). **e** Quantification for the total moved distance between WT and $cKI^{f/+}$ mice in the open field test (WT n=21; $cKI^{f/+}$ n=13). **f** Representative tracks of WT and $cKI^{f/+}$ mice in the elevated-plus maze test. **g** Quantification for the time spent in the closed arms between WT and $cKI^{f/+}$ mice in the elevated-plus maze test (WT n=19; $cKI^{f/+}$ n=14). **h** Quantification for the time spent in the open arms between WT and $cKI^{f/+}$ mice in the elevated-plus maze test (WT n=19; $cKI^{f/+}$ n=14). **i** Schematic illustration of the NOR test. Familiar object (FO), Novel object (NO). **j** Quantification for the percentage of preference for novel object in WT and $cKI^{f/+}$ mice (WT n=12; $cKI^{f/+}$ n=10). **k** IF for BrdU in adult WT and $cKI^{f/+}$ mice hippocampus. Scale bar, 50μm. **l** Quantification for the number of BrdU labeling cells in 10^3μm hippocampus (WT n=4; $cKI^{f/+}$ n=6). Two-tail unpaired T-test is used to analyze the data, n.s. (no significant difference), $P<0.05$ (*), $p<0.01$(**), $p<0.001$(***).

Supplementary Fig. 7

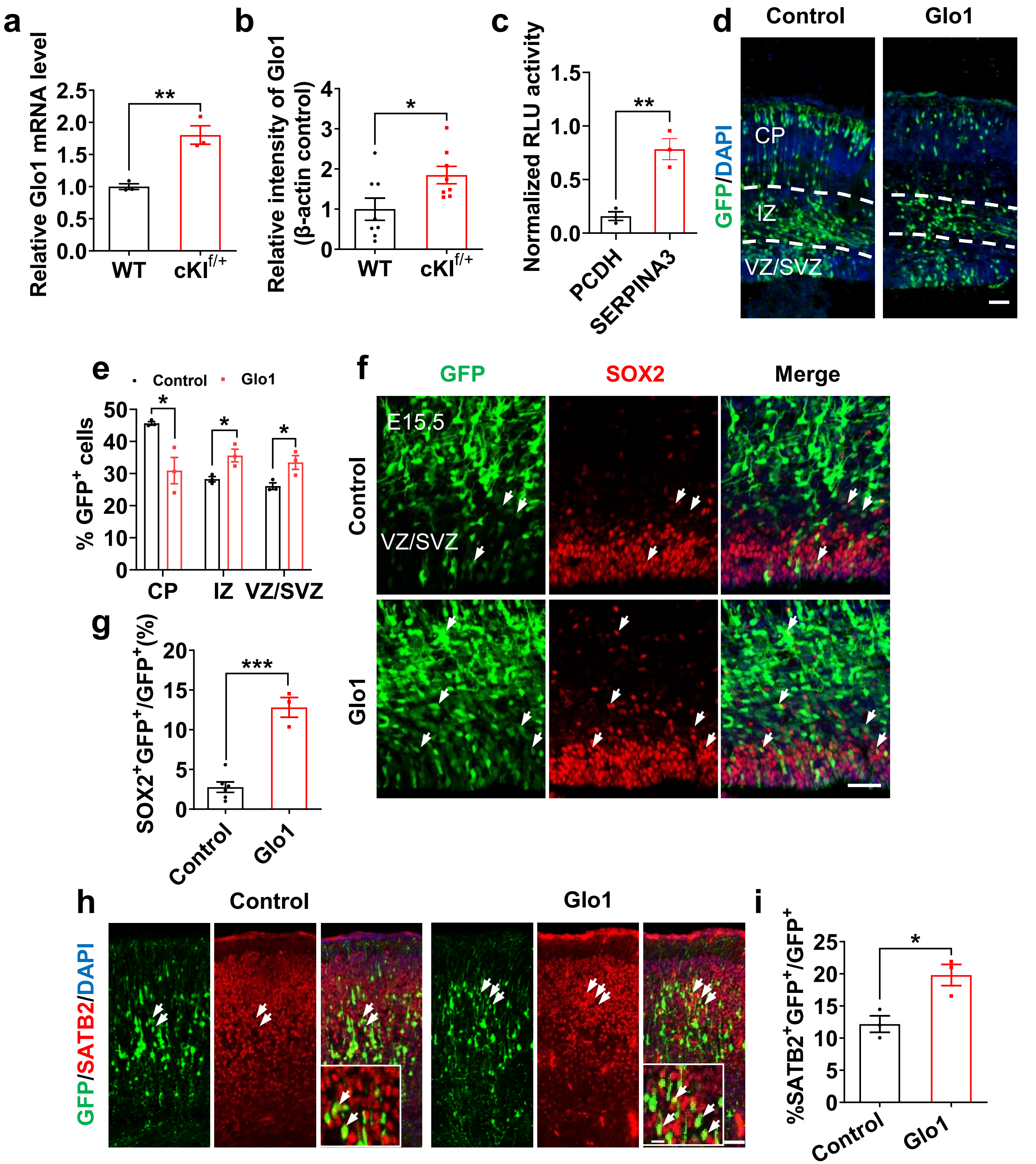

Supplementary Figure 7. Overexpression of Glo1 promotes the proliferation of NPCs and the increase of neurons. a RT-qPCR was performed to detect the mRNA level of Glo1 in WT and $cKI^{f/+}$ mouse cortices (n=3). **b** Statistics of relative intensity of Glo1 in western-blot measurement (n=8). **c** Quantification for the normalized RLU activity (n=3). **d** GFP-positive cell distribution in E15.5 mice after IUE at E13.5. Dashed line shows the boundary of different region. Scale bar, 50μm. **e** Quantification for the percentage of GFP^+ cells in different zone (n=3). **f** IF for SOX2 in E15.5 mice after IUE at E13.5. Arrows, GFP^+SOX2^+ cells. Scale bar, 50μm. **g** Quantification for the percentage of $SOX2^+$ cells among GFP^+ cells (control n=6; Glo1 n=3). **h** IF for SATB2 in P0 mice after IUE at E13.5. Higher magnification images show the $SATB2^+GFP^+$ cell (arrow, bottom left). Scale bars, 50μm, 10μm (bottom left). **i** Quantification for the percentage of $SATB2^+$ cells among GFP^+ cells (n=3). Two-tail unpaired T-test is used to analyze the data, n.s. (no significant difference), P<0.05 (*), p<0.01(**), p<0.001(***).

Supplementary Table S1. Marker genes for scRNA sequencing analysis.

Sheet 1

This table contains information of marker genes of all clusters.

Sheet 2

This table contains information of each cluster cell type identity.

Sheet 3

This table contains information of marker genes of each neuronal clusters.

Sheet 4

This table contains information of neuronal cell type of each cluster.

Supplementary Table S2. Primers used for RT-qPCR analysis.

Gene	Forward	Reverse
SERPINA3	GTGGTCCATAAGGCTGTGCT	GGCCTGTTGAAACGCACAAT
PAX6	CAGAACAGTCACAGCGGAGT	GTCTGATGGAGCCAGTCTCG
HOPX	TGCTCATTTTCCTGGGCTGT	CCAGGATTTCCACCTGGTCC
NESTIN	AGTGATGCCCCTTCACCTTG	GCTCGCTCTCTACTTTCCCC
SOX2	GGATAAGTACACGCTGCCCG	ATGTGCGCGTAACTGTCCAT
β-actin	CCGTTGCCCTGAGGCTCTTT	GGTCTTTGCGGATGTCCACG
Glo1	CCCTCGTGGATTTGGTCACA	AGCCGTCAGGGTCTTGAATG
Target (Glo1) site	Forward	Reverse
-5k	CATGGGTCTTTCCCAGTGACTT	GAGATGCGTGTGATGAACAAACA
-3k	TGAGTGAAAGAGACTATAGAGAGGC	GTGGAGAAACAATAAGAACACACA

-2.5k	AGTTTATCTCAAGCATTCACACTCT	AGTGCTGGGATGAAAGGCT
-2k	CCCATCCAATCCCCACTCTC	GCAGCCTGCTCCTCCAAAT
-1.5k	AAGCTGAGGGAAGGCAAGTC	ATTGCAGAGTCCATGCTGGT
-1k	GACAAAGCACCCGGAGTCATA	CTTTGTCCCCCAACAGGACC
-0.5k	TTGACAGGTCTCACCAGCAC	TGCTTTGGCGGTCTAAGGTT
CDS	CCCTCGTGGATTTGGTCACA	AGCCGTCAGGGTCTTGAATG

Supplementary Table S3. Key resources table.

REAGENT or RESOURCE	SOURCE	IDENTIFIER
**Antibodies**		
Rabbit anti-SERPINA3	Proteintech	Cat#12192-1-AP, RRID:AB_10344682
Mouse anti-SERPINA3	Proteintech	Cat#66078-1-Ig, RRID:AB_11182502
Rabbit anti-HOPX	Proteintech	Cat#11419-1-AP, RRID:AB_10693525
Rabbit anti-SOX2	Cell Signaling Technology	Cat# 3728, RRID:AB_2194037
Goat anti-SOX2	R&D	Cat# AF2018, RRID:AB_355110
Rabbit anti-PAX6	Millipore	Cat# AB2237, RRID:AB_1587367
Rat anti-TBR2	Thermo Fisher Scientific	Cat#14-4875-82, RRID:AB_11042577
Mouse anti-P-VIM	Abcam	Cat# ab22651, RRID:AB_447222
Mouse anti-P-VIM	MBL	Cat# D095-3, RRID:AB_592969
Rabbit anti-PH3	Cell Signaling Technology	Cat# 3377, RRID:AB_1549592
Rat anti-BrdU	Abcam	Cat# ab6326, RRID:AB_305426
Mouse anti-CUX1	Santa	Cat# F2515
Rabbit anti-CUX1	Proteintech	Cat# 11733-1-AP, RRID:AB_2086995
Mouse anti-SATB2	Abcam	Cat# ab51502, RRID:AB_882455
Rat anti-CTIP2	Abcam	Cat# ab18465, RRID:AB_2064130
Rabbit anti-TBR1	Abcam	Cat# ab31940, RRID:AB_2200219
Rabbit anti-TUJ1	Bioworld Technology	Cat# AP0013, RRID:AB_2797404
Rabbit anti-NEUN	Abcam	Cat# ab177487, RRID:AB_2532109
Mouse anti-MAP2	Millipore	Cat# MAB378, RRID:AB_94967

Mouse anti-β-Actin	Proteintech	Cat# 60008-1-Ig, RRID:AB_2289225
Rabbit anti-β-Actin	Proteintech	Cat#20536-1-AP, RRID:AB_10700003
Rat anti-GLO1	Sigma-Aldrich	Cat#SAB4200193, RRID:AB_10637845
Rabbit anti-Olig2	Abcam	Cat# ab109186, RRID:AB_10861310
Rabbit anti-S100β	Abcam	Cat# ab52642, RRID:AB_882426
Mouse anti-ALDH1L1	Abcam	Cat# ab56777, RRID:AB_940204
Rat anti-MBP	Millipore	Cat# MAB386, RRID:AB_94975
**Chemicals, Peptides, and Recombinant Proteins**		
5-Bromo-2-deoxyuridine (BrdU)	Sigma-Aldrich	Cat# B5002
4',6-Diamidino-2-Phenylindole, Dihydrochloride (DAPI)	Thermo Fisher	Cat# D1306; RRID: AB_2629482
5-ethynyl-20-deoxyuridine (EdU)	Thermo Scientific	Cat# C10639
SDS-RIPA lysis buffer	Bioteke	Cat# PP1901
Triton X-100	Sigma-Aldrich	Cat# T9284
fetal bovine serum	BIOCHROME	
Essential 8 medium	Thermo	Cat# A1517001
DMEM/F12	Invitrogen	Cat# 10565018
GlutaMAX	Invitrogen	Cat# 35050061
B27 (without VA)	Invitrogen	Cat# 12587-010
basic fibroblast growth factor	Invitrogen	Cat# 13256029
epidermal growth factor	Invitrogen	Cat# E3480
neurobasal medium	Invitrogen	Cat# A3582901
N2 supplement	Invitrogen	Cat# 17502048
dorsomorphin	Sigma	Cat# P5499-25MG
SB431542	Sigma	Cat# 616461-5MG
CHIR99021	Sigma	Cat# SML1046-5MG
**Experimental Models: Cell Lines**		
H9 human ES cells	WiCell Research	
Neuro 2a cell	American Type Culture Collection	
HEK293T	American Type Culture Collection	
**Experimental Models:Organisms/Strains**		
NestinCre:: SERPINA3$^{f/+}$	this paper	N/A
hSERPINA3 floxed mice	this paper	N/A
Nestin-Cre transgenic mice	The Jackson Laboratory	Cat#JAX:003771, RRID:IMSR_JAX:003771
**Critical Commercial Assays**		
Click-iT EdU Alexa Fluor 594	Invitrogen	Cat# C10339

Imaging Kit		
SuperReal PreMix Plus (SYBR Green) kit	TIANGEN	Cat# FP205-01
In Situ Cell Death Detection Kit	Roche	Cat# 11684795910
**Software and Algorithms**		
ZEN	ZEN Digital Imaging for Light Microscopy	RRID:SCR_013672
Graphpad Prism 8	Graphpad Software	RRID: SCR_002798
EthoVision XT	Noldus	RRID:SCR_000441
